# Predicting patient-reported and objectively measured functional outcome 6 months after ankle fracture in people aged 60 years or over in the UK: prognostic model development and internal validation

**DOI:** 10.1136/bmjopen-2019-029813

**Published:** 2019-07-23

**Authors:** David J Keene, Karan Vadher, Keith Willett, Dipesh Mistry, Matthew L Costa, Gary S Collins, Sarah E Lamb

**Affiliations:** 1 Nuffield Department of Orthopaedics, Rheumatology and Musculoskeletal Sciences, University of Oxford, Oxford, UK; 2 Warwick Clinical Trial Unit, University of Warwick, Coventry, UK; 3 Centre for Statistics in Medicine, University of Oxford, Oxford, UK

**Keywords:** ankle fractures, ankle injuries, osteoporotic fractures, prognosis

## Abstract

**Objective:**

To predict functional outcomes 6 months after ankle fracture in people aged ≥60 years using post-treatment and 6-week follow-up data to inform anticipated recovery, and identify people who may benefit from additional monitoring or rehabilitation.

**Design:**

Prognostic model development and internal validation.

**Setting:**

24 National Health Service hospitals, UK.

**Methods:**

Participants were the Ankle Injury Management clinical trial cohort (n=618) (ISRCTN04180738), aged 60–96 years, 459/618 (74%) female, treated surgically or conservatively for unstable ankle fracture. Predictors were injury and sociodemographic variables collected at baseline (acute hospital setting) and 6-week follow-up (clinic). Outcome measures were 6-month postinjury (primary) self-reported ankle function, using the Olerud and Molander Ankle Score (OMAS), and (secondary) Timed Up and Go (TUG) test by blinded assessor. Missing data were managed with single imputation. Multivariable linear regression models were built to predict OMAS or TUG, using baseline variables or baseline and 6-week follow-up variables. Models were internally validated using bootstrapping.

**Results:**

The OMAS baseline data model included: alcohol per week (units), postinjury EQ-5D-3L visual analogue scale (VAS), sex, preinjury walking distance and walking aid use, smoking status and perceived health status. The baseline/6-week data model included the same baseline variables, minus EQ-5D-3L VAS, plus five 6-week predictors: radiological malalignment, injured ankle dorsiflexion and plantarflexion range of motion, and 6-week OMAS and EQ-5D-3L. The models explained approximately 23% and 26% of the outcome variation, respectively. Similar baseline and baseline/6 week data models to predict TUG explained around 30% and 32% of the outcome variation, respectively.

**Conclusions:**

Predictive accuracy of the prognostic models using commonly recorded clinical data to predict self-reported or objectively measured ankle function was relatively low and therefore unlikely to be beneficial for clinical practice and counselling of patients. Other potential predictors (eg, psychological factors such as catastrophising and fear avoidance) should be investigated to improve predictive accuracy.

**Trial registration number:**

ISRCTN04180738; Post-results.

**Strengths and limitations of this study**

- Developed and internally validated prognostic models using robust methods.
- The Ankle Injury Management trial was a pragmatic study to enhance generalisability and had very low levels of missing data and is reflective of data that are or could be routinely collected during acute hospital admission and clinic follow-up.
- Use of an existing clinical trial dataset for developing the prognostic model restricted the choice of potential prognostic factors to those collected in the trial.

## Introduction

### Background

Ankle fractures in older people are increasing in number as the population ages.^1^ Older people have a worse prognosis than younger adults for recovering function after fractures.^2^ However, there is limited evidence on which injury, treatment and sociodemographic factors predict functional outcomes after ankle fracture in older adults. Previous studies have used small cohorts and few predictive factors.^3–5^

### Rationale

A multicentre randomised clinical trial of close contact casting versus surgery included 620 people aged 60 years and over with an unstable ankle fracture and found equivalence in outcomes between the treatment groups.^6 7^ Irrespective of the initial fracture treatment, considerable outcome variation was evident in the trial cohort, highlighting the value of investigating which combination of socio-demographic and clinical prognostic factors could predict functional outcomes in this patient group. A prognostic model could inform patient counselling about prognosis and identify people who might benefit from additional monitoring or rehabilitation.

### Objectives

Develop and internally validate a prognostic model to predict (1) patient-reported and (2) objectively measured functional outcome 6 months after ankle fracture in people aged 60 years or over using sociodemographic and clinical data collected in the acute phase and at 6-week follow-up.

## Methods

### Study design and setting

Data from the Ankle Injury Management (AIM) trial cohort (registration: ISRCTN04180738) were used to develop and internally validate prognostic models. AIM was a pragmatic, multicentre, randomised equivalence trial and economic evaluation comparing close contact casting with open surgical reduction and internal fixation in the treatment of unstable ankle fractures in people aged over 60 years. The trial methods and results have been published elsewhere.^6 7^ Participants were recruited from May 2004 in a pilot centre, then from 24 centres (general hospitals and major trauma centres) in the UK from July 2010 to November 2013. The primary endpoint was 6 month follow-up. All participants gave written informed consent for data to be used.

### Participants

Eligible participants were aged 60 years or over and had an acute, overtly unstable ankle fracture (displaced or clinically unstable). Participants with insulin-dependent diabetes mellitus, active leg ulceration, critical limb ischaemia, open fracture, serious concomitant disease, substantial cognitive impairment or ankle arthritis, or who were not fit for anaesthesia, were excluded. Participants were allocated to usual care (open reduction and internal fixation surgery) or a minimally padded close contact cast. Both treatments were conducted under anaesthesia by an orthopaedic surgeon.

### Outcome measures

#### Primary outcome

The primary outcome to be predicted was ankle function, measured using the Olerud and Molander Ankle Score (OMAS)^8^ 6 months after fracture. The OMAS is a widely used questionnaire to assess outcomes after ankle fracture, covering a range of symptoms and mobility limitations. It is measured on a 0–100 scale, with lower scores representing worse ankle function. Participants reported their OMAS at follow-up clinics or, if unable to attend, over the telephone. If the participant did not have sufficient dexterity to complete the questionnaire independently the researcher acted as scribe.

#### Secondary outcome

The secondary outcome to be predicted was the Timed Up and Go (TUG) test,^9^ an objective measurement of mobility conducted by a blinded outcome assessor. Participants were timed standing up from a chair, walking 8.6 m, turning and returning to sit in the start position. Participants’ ankles had a dressing applied to obscure the presence of lack of surgical incision scars. The TUG test is responsive, valid and reliable assessment of mobility in older adults that has been shown to be predictive of falls risk and functional decline.^10–12^

### Predictors

Predictor variables were selected based on clinical rationale and availability in the clinical trial dataset (table 1). Two models were produced for each outcome to investigate whether predictor variables collected at 6-week follow-up clinics improved the baseline prediction. Predictor measurements were obtained from participant questionnaires or clinical assessments. Two experienced orthopaedic surgeons with no access to the clinical data assessed fracture misalignment at 6 weeks on anteroposterior or mortise and lateral radiographs.

**Table 1:** Preselected predictors at baseline

	Measurement
Baseline predictors	
Age	Years
Sex	Male, female
Treatment received	Close contact cast/internal fixation surgery/other procedure
Fracture classification	Trans-infra-syndesmotic (Weber A and B), supra-syndesmotic (Weber C)
Health status	Excellent, Very good, Good, Fair, Poor
Smoking status	Never, Ex-smoker, Yes
Admitted from	Own home, warden accommodation, acute hospital, community hospital, temporary residence
Home support	Lives alone, lives with someone, lives with carer, home care package
Alcohol status	Units per week
Cognitive function: Mini-Mental State Examination (MMSE)	Score (0–30, higher scores indicate better cognitive function)
Number of comorbidities	≥0
Walking distance preinjury	About house, less than 100 m, less than 0.5 mile, more than 0.5 mile
Walking aid preinjury	None, one stick, two sticks, frame/rollator, wheelchair
Preinjury ankle function: Preinjury OMAS (recall of preinjury status)	Score (0–100, higher score indicates better ankle function)
Health-related quality of life (HRQL): EQ-5D-3L score at: Day before injury Postinjury (at baseline assessment) EQ-5D-3L visual analogue scale (VAS) at: Day before injury Postinjury (at baseline assessment)	EQ-5D-3L score (upper bound equal to one indicates full health, 0 represents death, negative scores indicate a state worse than death) EQ-5D-3L VAS (0–100, higher scores indicate better HRQL)
6-week follow-up predictors	
OMAS at 6 weeks	Score (0–100)
EQ-5D-3L VAS 6 week	As baseline
EQ-5D-3L score at 6 weeks after injury	As baseline
Injured ankle range of dorsiflexion	Hand-held goniometry, degrees
Injured ankle range of plantar flexion	Hand-held goniometry, degrees
Readmission to hospital	Yes/No
Started partial weight bearing by 6 weeks	Yes/No
Radiological malalignment in 6 week radiograph	Yes/No

*Radiological malalignment at 6 weeks was assessed by a combination of bespoke measurement software and verification by two experienced consultant surgeon using the criteria: radiograph demonstrating any one or combination of showing talar subluxation >2 mm (talar shift), excessive talar tilt (>2°), or a diastasis (tibiofibular clear space ≥5 mm).

OMAS, Olerud and Molander Ankle Score.

### Statistical analysis

Sample size was constrained to the size of the AIM trial cohort as this was a pre-existing dataset. The number of variables was limited to those plausibly related the outcome. The OMAS and TUG multivariable models were built using data from 620 participants. We initially assessed the covariates by plotting scatter graphs of outcomes and each continuous covariate and comparing each covariate with another. If highly correlated predictors were found, only one was included in the multivariable modelling. Any predictors or participants with >90% missing data were excluded. Remaining missing data were dealt with using single imputation before building the model.

As the outcomes were continuous, the prognostic models were developed under a linear regression modelling framework. The predictors (table 1) were included as independent variables in the model. Backwards elimination was performed to derive the models using the Akaike information criterion (AIC).

Categorical variables were collapsed into binary variables to ensure sufficient data points in each subcategory. The use of an assistive device for walking before injury was collapsed into whether the patient used assistance (yes: one stick, two sticks, frame/rollator, or wheelchair) or not (no). Walking distance before injury was split into <0.5 miles and >0.5 miles, reflecting the person’s walking endurance. Home support was split into lives alone/with someone or lives with carer/has external care support. Continuous variables were investigated for non-linearity with the outcome using multivariable fractional polynomials.

Clinically plausible interactions were examined for inclusion in the model. Model performance was assessed by calculating the adjusted R^2^.

The prognostic models were internally validated through bootstrapping using 200 bootstrap samples (replaying all variable selection procedures) to correct for optimism and quantify and adjust the model for overfitting.^13^ All analyses were conducted using Stata V.14.2. We followed the TRIPOD statement when reporting this study.^14^

### Patient and public involvement

Patient and public involvement (PPI) representatives provided feedback on the research proposal in the planning stages of this study. A PPI representative was an independent member of the AIM trial steering committee.

## Results

### Participants

The dataset contained 620 participants. Participants were aged median 70 (IQR 65–76) years and 459/618 (74%) were female. Tables 2 and 3 list the participant characteristics considered for building the models, the median (IQR) values for continuous variables, and the median OMAS and TUG values for categorical variables.

**Table 2:** Median (IQR) values for the baseline and 6 week continuous variables and the 6-month outcomes

Continuous variable	n	Median (IQR)
6-month OMAS score	592	70 (50–80)
6-month TUG	550	18 (14–23)
Age (years)	618	70 (65–76)
Mini Mental State Examination (MMSE)	556	29 (27–30)
Number of alcohol units consumed in a week	614	2 (0–10)
EQ-5D-3L VAS	557	85 (75–95)
EQ-5D-3L VAS postinjury	521	58 (40–75)
Number of comorbidities	618	1 (1–2)
Preinjury OMAS	618	100 (80–100)
EQ-5D-3L score	557	1 (0.80–1)
EQ-5D-3L score postinjury	522	0.02 (−0.06–0.16)
Injured ankle range dorsiflexion	578	5 (0–10)
Injured ankle range plantar flexion	578	20 (12–30)
6-week OMAS	605	35 (30–50)
6-week EQ-5D-3L score	550	0.52 (0.31–0.71)
6-week EQ-5D-3L VAS	550	75 (60–86)

OMAS, Olerud and Molander Ankle Score; TUG, Timed Up and Go; VAS, visual analogue scale.

**Table 3:** OMAS and TUG median (IQR) for baseline and 6-week categorical variables

Categorical variable	n	Median OMAS (IQR)	n	Median TUG (IQR)
Sex				
Male	151	75 (60–90)	142	17 (14–19)
Female	441	65 (48–80)	408	18 (15–24)
Smoking status				
Current smoker	55	65 (50–80)	51	18 (14–23)
Ex-smoker	234	68 (50–80)	216	18 (14–23)
Never	303	70 (50–80)	283	18 (14–23)
Health status				
Excellent	135	75 (60–85)	132	15 (13–19)
Very good	250	70 (55–85)	233	18 (14–21)
Good	153	60 (45–80)	142	20 (16–27)
Fair	50	53 (35–70)	41	24 (18–33)
Poor	4	48 (11–88)	2	44 (28–59)
Admitted from				
Own home	576	70 (50–80)	539	18 (14–23)
Warden accommodation	6	68 (41–86)	4	18 (17–25)
Acute hospital	2	55 (40–70)	2	29 (18–40)
Community hospital	2	75 (70–80)	1	14 (14–14)
Temporary residence	6	75 (59–91)	4	14 (12–20)
Home support				
Lives alone	188	70 (50–85)	173	19 (15–24)
Lives with someone	398	70 (50–80)	375	17 (14–22)
Lives with carer	1	35 (35–35)	1	34 (34–34)
Home care package	5	40 (25–55)	1	15 (15–15)
Walking aid preinjury				
None	512	70 (55–85)	491	17 (14–21)
One stick	58	45 (35–61)	50	26(21–32)
Two sticks	6	48 (30–65)	4	37 (18–59)
Frame/rollator	13	40 (25–70)	4	88 (40–192)
Wheelchair	3	70 (45–95)	1	46 (46–46)
Walking distance preinjury				
About house	17	55 (35–70)	6	29 (26–112)
Less than 100 m	33	40 (28–58)	28	37 (27–47)
Less than 0.5 mile	58	55 (40–75)	52	22 (17–28)
More than 0.5 mile	484	70 (55–85)	464	17 (14–21)
Fracture pattern				
Weber A and B	516	70 (50–80)	480	18 (14–23)
Weber C	76	65 (45–79)	70	18 (15–23)
Treatment received				
Close contact casting	271	70 (50–80)	246	18 (15–23)
Internal fixation surgery	308	70 (55–80)	292	17 (14–22)
Other	13	55 (38–85)	12	20 (17–29)
Readmission				
No	542	70 (50–80)	506	18 (14–23)
Yes	50	63 (50–75)	44	18 (14–21)
Started partial weight bearing				
No	224	70 (50–85)	213	17 (14–22)
Yes	368	70 (50–80)	337	18 (14–23)
Radiological malalignment				
No	365	70 (50–80)	343	18 (14–23)
Yes	227	65 (45–80)	207	18 (15–23)

OMAS, Olerud and Molander Ankle Score; TUG, Timed Up and Go.

### Missing data

Two participants had >90% missing data and were omitted from the analysis. Most predictors did not exceed 16% missing data. Most missing data occurred in the baseline EQ-5D-3L postinjury score. There were complete baseline, 6-week and outcome (OMAS and TUG) data for 434/620 (70%) participants.

### Predicting patient-reported functional outcome 6 months after ankle fracture

#### OMAS baseline model

Table 4 shows the eight baseline variables associated with 6 month OMAS and selected for the OMAS baseline model: preinjury OMAS, alcohol consumed per week (units), postinjury EQ-5D-3L visual analogue scale (VAS), sex, walking distance, walking aid, smoking status and health status. For instance, if all variables were kept constant, the 6-month OMAS for women was on average approximately 7 points lower than for equivalent men (p<0.001, 6-month OMAS median (IQR), 70 (50–80)). A 10-point difference in the preinjury OMAS for two identical individuals led to an average difference of approximately 2 points in the 6-month OMAS. The units of alcohol consumed in a week and the postinjury EQ-5D-3L VAS had marginal (p=0.043) and non-influential (p=0.102) effects on the 6-month OMAS, respectively. People who could walk further than 0.5 miles before injury had an approximately 8-point higher OMAS than those who could not, and people who required a walking aid before injury were more likely to have an approximately 8-point lower OMAS.

**Table 4:** Univariable analysis and the final baseline and baseline plus 6-week follow-up linear multivariable models for the 6 month OMAS score

Variable	Univariable analysis		Final multivariable baseline model		Final multivariable baseline plus 6-week follow-up model	
	β coefficient (95% CI)	P value	β coefficient (95% CI)	P value	β coefficient (95% CI)	P value
Baseline						
Constant			44.34 (30.04 to 58.64)	<0.001	34.62 (20.00 to 49.23)	<0.001
Preinjury OMAS score	0.48 (0.39 to 0.57)	<0.001	0.22 (0.09 to 0.34)	0.001	0.19 (0.07 to 0.31)	0.002
Alcohol consumed per week (units)	0.34 (0.16 to 0.52)	<0.001	0.14 (−0.03, 0.32)	0.102	0.23 (0.07 to 0.40)	0.007
EQ-5D-3L VAS Score (postinjury)	0.18 (0.10 to 0.25)	<0.001	0.08 (0.003 to 0.15)	0.043		
Sex		<0.001		<0.001		0.010
Female	−8.94 (–12.82 to 5.05)		−6.90 (−10.75 to 3.06)		−4.99 (−8.79 to 1.19)	
Walking distance*		<0.001		0.002		0.015
>0.5 mile	19.10 (15.01 to 23.19)		7.79 (2.97 to 12.61)		5.90 (1.16 to 10.64)	
Walking aid		<0.001		0.003		0.006
Yes	−20.31 (–24.95, to 15.66)		−8.35 (−13.89, to 2.82)		−7.60 (13.01 to 2.20)	
Smoking status†		0.185		0.049		0.061
Ex-smoker	−1.57 (−7.87, 4.72)		−3.31 (−8.93, 2.30)		−3.26 (−8.73, 2.20)	
Never	1.83 (−4.33, 8.00)		0.85 (−4.69, 6.38)		0.62 (−4.76, 6.00)	
Health status‡		<0.001		0.023		0.050
Very good	−4.19 (−8.50, 0.13)		−1.79 (−5.82, 2.24)		−2.25 (−6.17, 1.66)	
Good	−13.42 (−18.13 to 8.70)		−6.90, (−11.48 to 2.32)		−6.50 (−10.93 to 2.07)	
Fair	−20.69 (−27.41 to 13.97)		−2.97 (−10.32, 4.38)		−4.48 (−11.51, 2.56)	
Poor	−23.48 (−44.30to 2.66)		5.94 (−14.25, 26.14)		2.95 (−16.61, 22.51)	
Patient age	−0.54 (−0.77 to 0.31)	<0.001				
MMSE	1.21 (0.43 to 2.00)	0.003				
EQ-5D-3L VAS Score (day before injury)	0.35 (0.25 to 0.46)	<0.001				
EQ-5D-3L score (day before injury)	32.3 (23.07 to 41.53)	<0.001				
EQ-5D-3L score (postinjury)	11.24 (4.65 to 17.83)	<0.001				
Number of comorbidities	−3.58 (−4.89 to 2.28)	<0.001				
Fracture pattern		0.175				
Weber C	−3.59 (−8.78, 1.60)					
Admitted from§		0.441				
Warden accommodation	−8.31 (−23.57, 6.96)					
Acute hospital	−10.01 (−40.38, 20.37)					
Community hospital	9.99 (−20.38, 40.37)					
Temporary residence	10.96 (−5.35, 27.26)					
Treatment received¶		0.249				
ORIF	1.67 (−1.83, 5.18)					
Other	−6.33 (−16.75, 4.09)					
Home support **		0.003				
Live alone or with someone	26.11 (8.63 to 43.59)					
Additional 6 week variables						
6 week EQ-5D-3L VAS	0.32 (0.21 to 0.42)	<0.001				
Readmission to hospital						
Yes	−3.30 (−9.49, 2.89)	0.295				
Started partial weight bearing		0.575				
Yes	−1.01 (−4.54, 2.52)					
Radiological malalignment present		0.124				0.025
Yes	−2.78 (−6.33, 0.77)				−3.54 (−6.62 to 0.45)	
Range of injured ankle motion Dorsiflexion	0.15 (−0.04, 0.34)	0.131			0.18 (0.007 to 0.35)	0.041
Range of injured ankle motion Plantar flexion	0.14 (0.01 to 0.28)	0.032			0.17 (0.05 to 0.28)	0.006
6 week OMAS	0.33 (0.22 to 0.44)	<0.001			0.19 (0.09 to 0.30)	<0.001
6 week EQ-5D-3L score	25.69 (18.66 to 32.72)	<0.001			10.21 (3.64 to 16.78)	0.002

*Reference category for walking distance is ‘<0.5 mile’.

†Reference category for smoking status is ‘Current smoker’.

‡Reference category for health status is ‘Excellent’.

§Reference category for admitted from is ‘Own home’.

¶Reference category for treatment received is ‘CCC’.

**Reference category for home support is ‘live with care or has external care support’.

NB, EQ-5D-3L VAS Score (postinjury) omitted from final model by backward selection and correlation with 6-week score.

MMSE, Mini-Mental State Examination; OMAS, Olerud and Molander Ankle Scale; ORIF, Open Reduction and Internal Fixation; VAS, Visual Analogue Scale.

The initial model containing all candidate predictors had an R^2^ value of 0.25. The final model had an R^2^ value of 0.238 and an adjusted R^2^ of 0.223: the model’s predictors explained approximately 22% of the outcome variation.

#### OMAS baseline model internal validation

After bootstrapping, the optimism-corrected R^2^ performance estimate was 0.228. The model’s performance on the internal validation was similar to that achieved on the original dataset, indicating no evidence for any overfitting.

#### OMAS baseline/6-week model

Adding the 6-week follow-up variables produced a model similar to the baseline model, minus postinjury EQ-5D-3L VAS, plus five 6-week follow-up variables: presence of radiological malalignment, range of motion in the injured ankle for dorsiflexion and plantar flexion, 6-week OMAS and 6-week EQ-5D-3L (table 4). A patient with radiological malalignment at 6 weeks would, on average, have a 6-month OMAS approximately 4 points lower than a patient without malalignment. Keeping all other variables constant, 20° more ankle motion in both dorsiflexion and plantar flexion at 6 weeks would result in an approximately 4-point higher 6-month OMAS. The higher the 6-week OMAS and EQ-5D-3L, the greater the 6-month OMAS (p<0.001 and p=0.002, respectively).

The baseline/6-week model had an adjusted R^2^ value of 0.261 which was higher than the baseline model’s adjusted R^2^. Including the 6-week follow-up variables explained around 4% more variation than the baseline model.

#### OMAS baseline/6-week model internal validation

After bootstrapping, the optimism-corrected R^2^ performance estimate was 0.264, similar to that on the original dataset. Approximately 26% of the variation in the 6 month OMAS was explained by the model, an increase of 3% from the baseline linear multivariable model.

In an exploratory analysis, a model using only the 6-week variables was developed. The adjusted R^2^ value was 0.123, which was significantly lower than the model performance for the baseline model.

### Predicting objectively measured functional outcome 6 months after ankle fracture

#### TUG baseline model

Table 5 shows the baseline variables associated with the TUG time at 6 months and included in the baseline TUG model. On average women had around 6 s (p<0.001) longer TUG times than men (6-month TUG median (IQR), 18 s (14 to23)). Holding all other variables constant, a 10-point increase in the mini-mental state examination score led to a 2 s decrease in the predicted TUG time (p<0.001). A 10-year age difference between two otherwise identical individuals resulted in an 8 s longer predicted TUG time for the older individual (p<0.001). Those with higher postinjury EQ-5D-3L VAS were more likely to have a quicker TUG time at 6 months (p<0.001). People able to walk further than 0.5 mile before injury had a TUG time approximately 20 s quicker than an equivalent person who could only walk less than 0.5 mile (p<0.001). People who required a walking aid before injury were 19 s slower, on average, than those who did not. Fracture pattern and preinjury OMAS predicted the TUG score well: a Weber C fracture pattern and low preinjury OMAS predicted a longer TUG time.

**Table 5:** Univariable analysis and the final baseline and baseline plus 6-week follow-up linear multivariable models for the 6month TUG score

Variable	Univariable analysis		Final multivariable baseline model		Final multivariable baseline plus 6-week follow-up model	
	β coefficient (95% CI)	P value	β coefficient (95% CI)	P value	β coefficient (95% CI)	P value
Baseline						
Constant			24.86 (−0.31, 50.03)	0.05	51.72 (29.34 to 74.11)	<0.001
Preinjury OMAS score	−0.38 (−0.45 to 0.30)	<0.001	−0.09 (−0.19, 0.0007)	0.05		
Alcohol consumed per week (units)	−0.24 (−0.38 to 0.10)	0.001				
EQ-5D-3L VAS Score (postinjury)	−0.12 (−0.18 to 0.06)	<0.001	−0.05 (−0.10, 0.006)	0.08		
Sex		<0.001		0.008		0.02
Female	5.64 (2.51 to 8.78)		3.61 (0.93 to 6.29)		3.30 (0.57 to 6.03)	
Walking distance*		<0.001		<0.001		<0.001
>0.5 mile	−19.67 (−22.80 to 16.53)		−12.26 (−15.87 to 8.65)		−9.90 (−13.30 to 6.50)	
Walking aid		<0.001		0.001		<0.001
Yes	19.00 (15.35 to 22.64)		7.67 (3.34 to 11.99)		6.10 (2.35 to 9.85)	
Smoking status†		0.33				0.04
Ex-smoker	−0.06 (−5.11, 4.99)				−1.26 (−5.48, 2.96)	
Never	−2.15 (−7.10, 2.81)				−3.93 (−8.07, 0.21)	
Health status‡		<0.001				
Very good	1.62 (−1.84, 5.07)					
Good	7.68 (3.90 to 11.46)					
Fair	18.08 (12.70 to 23.47)					
Poor	23.27 (6.60 to 39.94)					
Patient age	0.79 (0.61 to 0.97)	<0.001	0.36 (0.18 to 0.54)	<0.001	0.37 (0.20 to 0.54)	<0.001
MMSE	−2.15 (−2.75 to 1.54)	<0.001	−0.89 (−1.46 to 0.32)	0.002	−1.35 (−1.91 to 0.79)	<0.001
EQ-5D-3L VAS Score (day before injury)	−0.35 (−0.43 to 0.26)	<0.001				
EQ-5D-3L score (day before injury)	−21.22 (−28.46 to 13.97)	<0.001	6.73 (−0.99, 14.44)	0.09		
EQ-5D-3L score (postinjury)	−8.48 (−13.92 to 3.03)	0.002				
Number of comorbidities	3.43 (2.34 to 4.46)	<0.001				
Fracture pattern		0.03		0.03		
Weber C	4.61 (0.46 to 8.76)		3.97 (0.44 to 7.50)			
Admitted from§		0.67				
Warden accommodation	0.82 (−11.44, 13.07)					
Acute hospital	6.72 (−17.66, 31.11)					
Community hospital	−0.50 (−24.89, 23.89)					
Temporary residence	−9.53 (−22.62, 3.56)					
Treatment received¶		0.35				
ORIF	−0.94 (−3.75, 1.87)					
Other	4.95 (−3.41, 13.31)					
Home support**		0.03				
Live alone or with someone	−15.40 (−29.47 to 1.34)					
Additional 6 week variables						
6-week EQ-5D-3L VAS	−0.24 (−0.32 to 0.16)	<0.001			−0.07 (−0.14, 0.00)	0.05
Readmission to hospital		0.06				0.03
Yes	−4.83 (−9.81, 0.15)				−4.49 (−8.60 to 0.38)	
Started partial weight bearing		0.96				
Yes	0.08 (−2.77, 2.92)					
Radiological malalignment present		0.24				
Yes	−1.73 (−4.59 1.13)					
Range of injured ankle motion dorsiflexion	−0.005 (−0.16, 0.15)	0.95				
Range of injured ankle motion plantar flexion	0.01 (−0.09, 0.12)	0.83				
6-week OMAS	−0.13 (−0.22 to 0.04)	0.005				
6-week EQ-5D-3L score	−23.76 (−29.46 to 18.06)	<0.001			−11.11 (−16.13 to 6.09)	<0.001

*Reference category for walking distance is ‘<0.5 mile’.

†Reference category for smoking status is ‘Current smoker’.

‡Reference category for health status is ‘Excellent’.

§Reference category for admitted from is ‘Own home’.

¶Reference category for treatment received from is ‘CCC’.

**Reference category for home support is ‘live with care or has external care support’.

NB, EQ-5D-3L VAS Score (postinjury) omitted from final model by backward selection and correlation with 6-week score.

MMSE, Mini-Mental State Examination; OMAS, Olerud and Molander Ankle Scale; ORIF, Open Reduction and Internal Fixation; VAS, Visual Analogue Scale

The initial model containing all possible variables had an R^2^ value of 0.321, indicating that all of the variables chosen for consideration in model building together explained 32% of the variation in the TUG outcome. The final baseline model had an R^2^ value of 0.308 and an adjusted R^2^ of 0.298, indicating that the model’s predictors explained approximately 30% of the outcome variation.

#### TUG baseline model internal validation

After bootstrapping, the optimism-corrected R^2^ performance estimate was 0.293. The model’s performance on the internal validation was very similar to that achieved on the original dataset, explaining approximately 30% of the variation in the 6 month TUG score.

#### TUG baseline/6-week model

When the 6-week variables were included in building a model to predict the 6-month TUG time, four baseline variables were removed. The model-building process selected another baseline variable, home support, for inclusion in their place. Its inclusion led to spurious results as there were little data in one of the subcategories (lives with carer/has external care support). The variable was therefore removed from the final model. The final model also included three 6-week variables: readmission, 6-week EQ-5D-3L and 6-week EQ-5D-3L VAS (table 5).

If a person was readmitted in the first 6 weeks after injury, they were predicted to have a 4 s faster TUG time at 6 months (p=0.04), if all other variables were held constant. Those with a better EQ-5D-3L score at 6 weeks were more likely to have a quicker TUG (p<0.001). If all other variables were kept constant, an increase of 20 points in the 6-week VAS resulted in a 1.5 s quicker time (p=0.04).

The baseline/6-week data model had a greater adjusted R^2^ (0.321) than the baseline data model (0.308). The model now explained 32% of the variability in TUG at 6 months, 2% more than that explained by the baseline data model.

#### TUG baseline/6-week model internal validation

After bootstrapping, optimism-corrected R^2^ performance estimate was 0.314. The model’s performance on the internal validation dataset was very similar to that achieved on the original dataset, explaining approximately 31% of the variation in the 6 month TUG time.

In an exploratory analysis, a model using only the 6 week variables was developed. The adjusted R^2^ value was 0.109, which was lower than the model performance for the baseline model.

### Sensitivity analysis

By using single imputation for the missing values in the 6-month OMAS and TUG outcomes, we assumed that the missing values occurred at random, so that variables in the dataset could predict the occurrence of missing values. To check how sensitive the four models were to this assumption, we conducted a complete-case analysis, which assumes that missing values occur completely at random.

Twenty-six participants were omitted from the OMAS models, leaving 592 participants. The same variables were selected for the baseline data model. The same variables were selected for the baseline/6 week data model, plus postinjury EQ5D-3L VAS and ‘started partial weight bearing’. Both models had marginally lower adjusted R^2^ values: 0.205 for the baseline data model, 2% lower than the original model, and 0.256 for the baseline/6-week data model, 0.5% lower than the original model. After bootstrapping, the optimism-corrected R^2^ performance estimates of 0.215 for the baseline data model and 0.268 for the baseline/6-week data model.

Sixty-eight participants were omitted from the TUG models, leaving 550 participants. The baseline data model included the same variables as before, but replaced baseline postinjury EQ-5D-3L VAS with baseline recall preinjury EQ-5D-3L VAS and omitted preinjury OMAS. The baseline/6-week data model included the same variables as before, plus fracture pattern and walking aid. Both models had lower adjusted R^2^ values: 0.222 for the baseline data model, 8% lower than the original model, and 0.264 for the baseline/6-week data model, 6% lower than the original model. After bootstrapping, the optimism-corrected R^2^ performance estimates were 0.215 for the baseline data model and 0.253 for the baseline/6-week data model.

## Discussion

A prognostic model has the potential to inform anticipated recovery, and identify people who may benefit from additional monitoring or rehabilitation. We developed and internally validated four prognostic models for 6-month outcomes after ankle fracture using recommended methods^13^ and a wide range of regularly collected data on plausible prognostic factors from hospital admission and at 6 week follow-up clinics. The OMAS baseline data model included: alcohol per week (units), postinjury EQ-5D-3L VAS, sex, preinjury walking distance and walking aid use, smoking status and perceived health status. The baseline/6-week data model included the same baseline variables, minus EQ-5D-3L VAS, plus five 6-week predictors: radiological malalignment, injured ankle dorsiflexion and plantarflexion range of motion and 6-week OMAS and EQ-5D-3L. The models explained approximately 23% and 26% of the outcome variation, respectively. Similar baseline and baseline/6-week data models to predict TUG explained around 30% and 32% of the outcome variation, respectively. Adding 6-week follow-up variables to baseline variables provided only a modest benefit. This benefit arguably does not outweigh the logistical issues of obtaining patient information at 6 weeks to improve prediction accuracy. The models performed similarly on the development dataset and bootstrapped internal validation datasets.

Predictive accuracy of the four models using commonly recorded clinical data to predict self-reported or objectively measured ankle function 6 months after unstable ankle fracture in adults aged over 60 years was relatively low. As there are limitations in predictive performance, we do not recommend using these prognostic models in clinical practice as decision-making tools in isolation.

The variables of most predictive value across the prognostic models were sex, with females having worse outcomes than males. A cohort of 584 severe ankle sprain participants also found that females had worse outcomes.^15^ Self-reported preinjury walking distance and preinjury walking aid use was also predictive of outcome, with those able to walk less than 0.5 miles doing and using a walking aid faring worse than those able to walk further and not needing aids. These factors are features of declining locomotor function and are likely to be related to a greater levels of frailty preinjury.^16^

The inferences presented here cannot be extended to propose causal relationships between the predictors and outcomes, as we did not consider issues such as confounding. As the aim and design of this study prioritised optimising prognostic accuracy, predictors were considered without considering whether they could form a causal pathway or not.^17^

Comparisons with other studies that have developed prognostic models for people after ankle fracture are challenging as, to the best of our knowledge, this is the first study to focus on older adults, use a larger cohort, and internally validate the developed models to adjust for optimism. A two-centre observational study of 60 adults (mean age 49) reported that ankle dorsiflexion and fracture classification (number of malleoli injured) predicted OMAS 6 months after cast removal (R^2^=0.4).^3^ In a larger cohort of 150 adults (mean age 46), Lin and colleagues^7^ developed a model to predict patient-reported lower limb function (Lower Extremity Functional Scale)^18^ at 4 and 12 weeks after cast removal. The models were not internally validated, but were externally validated in a separate cohort of 94 participants. Eight predictors were examined. Pain and dorsiflexion after cast removal were included in the final model, which explained about 15% of the outcome variability in the development stage and even less in the external validation.

The lack of predictive power of both our models and those in the literature indicate that functional outcomes after ankle fracture may be difficult to predict, or other prognostic factors may need to be considered to improve predictions of functional outcome. Our models may be missing informative predictors that were not captured in the AIM trial dataset. More detailed injury characteristics, such as whether fractures were uni-malleolar, bi-malleolar or tri-malleolar, were not explored. The extent of articular damage assessed by specialist imaging could provide useful prognostic information. However, any techniques using specialist equipment or personnel would hamper clinical utility for routine use and would be difficult to implement if there were resource implications. Further research is recommended to investigate whether a wider range of psychosocial and environmental factors^19^ can enhance predictive accuracy. There is preliminary evidence that catastrophising and fear avoidance are potential psychological prognostic factors that warrant further investigation in people after ankle fracture.^20^

Similar results were found for the main and sensitivity analyses of both the OMAS and TUG models. The assumption that data were missing at random was therefore plausible and conducting single imputation before model building was probably appropriate.

This study was limited by the use of an existing clinical trial dataset for developing the prognostic model, as the choice of potential prognostic factors were restricted to those collected in the trial. Clinical trial cohorts are usually more selective than the wider clinical population. However, the AIM trial was a pragmatic study to enhance external validity. The use of a clinical trial cohort to develop prognostic models is also not uncommon, and has resulted in robust prognostic models for other ankle injury populations that have been successfully externally validated.^21^ Strengths of using this clinical trial dataset were that it had very low levels of missing data and that it reflected data that are or could be routinely collected during acute hospital admission and clinic follow-up.

## Conclusion

We developed and internally validated prognostic models to predict functional outcomes 6 months after unstable ankle fracture in older adults using commonly recorded clinical data. These prognostic models had relatively limited accuracy in predicting self-reported or objectively measured ankle function. Other potential predictors (eg, psychological factors such as catastrophising and fear avoidance) should be investigated.

## Supplementary Material

Reviewer comments

Author's manuscript
