# Supplementary material for: Predicting patient-reported and objectively measured functional outcome 6 months after ankle fracture in people aged 60 years or over in the UK: prognostic model development and internal validation
Source: BMJ Open. 2019 Jul 23;9(7):e029813. doi: 10.1136/bmjopen-2019-029813 (PMC6661636; doi:10.1136/bmjopen-2019-029813)
Supplement: Author's manuscript [file bmjopen-2019-029813.draft_revisions.pdf]

BMJ Open is committed to open peer review. As part of this commitment we make the peer review history of every article we publish publicly available.

When an article is published we post the peer reviewers' comments and the authors' responses online. We also post the versions of the paper that were used during peer review. These are the versions that the peer review comments apply to.

The versions of the paper that follow are the versions that were submitted during the peer review process. They are not the versions of record or the final published versions. They should not be cited or distributed as the published version of this manuscript.

BMJ Open is an open access journal and the full, final, typeset and author-corrected version of record of the manuscript is available on our site with no access controls, subscription charges or pay-per-view fees (http://bmjopen.bmj.com).

If you have any questions on BMJ Open's open peer review process please email info.bmjopen@bmj.com

BMJ Open

Prognostic Model Development and Internal Validation to Predict Function Six Months After Ankle Fracture in Older Adults

Journal:	*BMJ Open*
Manuscript ID	bmjopen-2019-029813
Article Type:	Research
Date Submitted by the Author:	12-Feb-2019

Complete List of Authors:	Keene, David; University of Oxford Nuffield Department of Orthopaedics Rheumatology and Musculoskeletal Sciences,<br>Vadher, Karan; University of Oxford Nuffield Department of Orthopaedics Rheumatology and Musculoskeletal Sciences<br>Willett, Keith; University of Oxford, Nuffield Department of Orthopaedics, Rheumatology and Musculoskeletal Sciences<br>Mistry, Dipesh; University of Warwick, Warwick Clinical Trial Unit<br>Costa, Matthew; University of Oxford, Nuffield Department of Orthopaedics, Rheumatology, and Musculoskeletal Sciences<br>Collins, Gary; University of Oxford, Centre for Statistics in Medicine<br>Lamb, Sarah; University of Oxford, Ndorms

Keywords:	ankle fractures, ankle injuries, osteoporotic fractures, prognosis

SCHOLARONE™
Manuscripts

Prognostic Model Development and Internal Validation to Predict Function Six Months After Ankle Fracture in Older Adults

David J. Keene, D.Phil[1,2]

Karan Vadher, MSc[1]

Keith Willett, FRCS, MBBS[1]

Dipesh Mistry, PhD[3]

Matthew Costa, FRCS, PhD[1.2]

Gary S. Collins, PhD[1]

Sarah E. Lamb, D.Phil[1,2]

[1]Nuffield Department of Orthopaedics, Rheumatology and Musculoskeletal Sciences, University of Oxford, Oxford, UK.

[2]Oxford NIHR Biomedical Research Centre, Oxford University Hospitals NHS Foundation Trust, UK.

[3]Warwick Clinical Trials Unit, University of Warwick, Coventry, UK.

Address correspondence to David Keene, Kadoorie Research Centre, John Radcliffe Hospital, Oxford, OX3 9DU, UK

E-mail: david.keene@ndorms.ox.ac.uk

Word count: 3550

For peer review only

Abstract

Objective: To predict functional outcomes 6 months after ankle fracture in people aged ≥60 years using post-treatment and 6-week follow-up data to inform anticipated recovery, and identify people who may benefit from additional monitoring or rehabilitation.

Design: Prognostic model development and internal validation.

Setting: 24 National Health Service hospitals, UK.

Methods: Participants: 618 AIM clinical trial participants (ISRCTN04180738), aged 60–96 years, 459/618 (74%) female, treated surgically or conservatively for unstable ankle fracture.

Predictors: Injury and socio-demographic variables collected at baseline (acute hospital setting) and 6-week follow-up (clinic).

Outcome measures: 6-month post-injury (primary) self-reported ankle function, using the Olerud and Molander Ankle Score (OMAS), and (secondary) Timed Up and Go (TUG) test by blinded assessor.

Missing data: Single imputation.

Statistical analysis: Multivariable linear regression models were built to predict OMAS or TUG, using baseline variables or baseline and 6-week follow-up variables. Models were internally validated using bootstrapping.

Results: The OMAS baseline data model included: alcohol per week (units), post-injury EQ-5D-3L VAS, sex, pre-injury walking distance and walking aid use, smoking status, and perceived health status. The baseline/6-week data model included the same baseline variables, minus EQ-5D-3L VAS, plus five 6-week predictors: radiological malalignment, injured ankle dorsiflexion and plantarflexion range of motion, and 6-week OMAS and EQ-5D-3L. The models explained approximately

For peer review only

23% and 26% of the outcome variation, respectively. Similar baseline and baseline/6-week data models to predict TUG explained around 30% and 32% of the outcome variation, respectively.

Conclusions: Prognostic models using commonly recorded clinical data have some ability to predict self-reported or objectively measured ankle function. Other potential predictors (e.g., psychosocial factors) should be investigated to improve predictive accuracy.

For peer review only

Key words: ankle fractures, ankle injuries, osteoporotic fractures, prognosis

Strengths and limitations of this study

- Developed and internally validated prognostic models using robust methods.
- Use of an existing clinical trial dataset for developing the prognostic model restricted the choice of potential prognostic factors to those collected in the trial.
- The AIM trial was a pragmatic study to enhance external validity and had very low levels of missing data and is reflective of data that is or could be routinely collected during acute hospital admission and clinic follow-up.
- Reported in accordance with the TRIPOD statement reporting guidance.

For peer review only

INTRODUCTION

Background

Ankle fractures in older people are increasing in number as the population ages.[1] Older people have a worse prognosis than younger adults for recovering function after fractures.[2] However, there is limited evidence on which injury, treatment, and socio-demographic factors predict functional outcomes after ankle fracture in older adults. Previous studies have used small cohorts and few predictive factors.[3-5]

Rationale

Considerable outcome variation was evident in a multicentre randomised clinical trial of 620 people aged 60 years or over who received surgery or a close contact cast for unstable ankle fracture.[6 7] This variability highlighted the value of investigating which combination of prognostic factors can predict functional outcomes. A prognostic model could inform patient counselling about prognosis and identify people who might benefit from additional monitoring or rehabilitation.

Objectives

Develop and internally validate a prognostic model to predict (1) patient-reported and (2) objectively measured functional outcome 6 months after ankle fracture in people aged 60 years or over using socio-demographic and clinical data collected in the acute phase and at 6-week follow-up.

For peer review only

METHODS

Study design and setting

Design Data from the Ankle Injury Management (AIM) trial cohort (registration: ISRCTN04180738) were used to develop and internally validate prognostic models. AIM was a pragmatic, multicentre, randomised equivalence trial and economic evaluation comparing close contact casting with open surgical reduction and internal fixation in the treatment of unstable ankle fractures in people aged over 60 years. The trial methods and results have been published elsewhere.[6 7] Participants were recruited from May 2004 in a pilot centre, then from 24 centres (general hospitals and major trauma centres) in the UK from July 2010 to November 2013. The primary endpoint was 6-month follow-up. All participants gave written informed consent for data to be used.

Participants

Eligible participants were aged 60 years or over and had an acute, overtly unstable ankle fracture. Participants with insulin-dependent diabetes mellitus, active leg ulceration, critical limb ischaemia, open fracture, serious concomitant disease, substantial cognitive impairment, or ankle arthritis, or who were not fit for anaesthesia, were excluded. Participants were allocated to usual care (open reduction and internal fixation surgery) or a minimally padded close contact cast. Both treatments were conducted under anaesthesia by an orthopaedic surgeon.

Outcome measures

Primary outcome: The primary outcome to be predicted was ankle function, measured using the Olerud and Molander Ankle Score (OMAS)[8] 6 months after

For peer review only

fracture. The OMAS is a widely used questionnaire to assess outcomes after ankle fracture, covering a range of symptoms and mobility limitations. It is measured on a 0 to 100 scale, with lower scores representing worse ankle function. Participants reported their OMAS at follow-up clinics or, if unable to attend, over the telephone.

Secondary outcome: The secondary outcome to be predicted was the Timed Up and Go (TUG) test,[9] an objective measurement of mobility conducted by a blinded outcome assessor. Participants were timed standing up from a chair, walking 8.6 m, turning, and returning to sit in the start position. Surgical incision sites on participants' ankles were obscured with a dressing to facilitate blinding.

Predictors

Predictor variables were selected based on clinical rationale and availability in the clinical trial dataset (**TABLE 1**). Two models were produced for each outcome to investigate whether predictor variables collected at 6-week follow-up clinics improved the baseline prediction. Predictor measurements were obtained from participant questionnaires or clinical assessments. Two experienced orthopaedic surgeons with no access to the clinical data assessed fracture misalignment at 6 weeks on anteroposterior or mortise and lateral radiographs.

Statistical analysis

Sample size was constrained to the size of the AIM trial cohort as this was a pre-existing dataset. The number of variables was limited to those plausibly related the outcome. The OMAS and TUG multivariable models were built using data from 620 participants. We initially assessed the covariates by plotting scatter graphs of

For peer review only

outcomes and each continuous covariate and comparing each covariate with another. If highly correlated predictors were found, only one was included in the multivariable modelling. Any predictors or participants with >90% missing data were excluded. Remaining missing data were dealt with using single imputation before building the model.

As the outcomes were continuous, the prediction models were developed under a linear regression modelling framework. The predictors (**TABLE 1**) were included as independent variables in the model. Backwards elimination was performed to derive the models using the Akaike information criterion (AIC).

Categorical variables were collapsed into binary variables to ensure sufficient data points in each subcategory. The use of an assistive device for walking before injury was collapsed into whether the patient used assistance (yes: one stick, two sticks, frame/rollator, or wheelchair) or not (no). Walking distance before injury was split into <0.5 miles and >0.5 miles, reflecting the person's walking endurance. Home support was split into lives alone/with someone or lives with carer/has external care support. Continuous variables were investigated for nonlinearity with the outcome using multivariable fractional polynomials.

Clinically plausible interactions were examined for inclusion in the model. Model performance was assessed by calculating the adjusted R2.

The prognostic models were internally validated through bootstrapping using 200 bootstrap samples (replaying all variable selection procedures) to correct for

For peer review only

optimism and quantify and adjust the model for overfitting.[10] All analyses were conducted using Stata 14.2. We followed the TRIPOD statement when reporting this study.[11]

Patient and Public Involvement

Patient and public involvement (PPI) representatives provided feedback on the research proposal in the planning stages of this study. A PPI representative was an independent member of the AIM trial steering committee.

For peer review only

RESULTS

Participants

The dataset contained 620 participants. Participants were aged median 70 (IQR 65 – 76) years and 459/618 (74%) were female. **TABLES 2 and 3** list the participant characteristics considered for building the models, the median [IQR] values for continuous variables, and the median OMAS and TUG values for categorical variables.

Missing data

Two participants had >90% missing data and were omitted from the analysis. Most predictors did not exceed 16% missing data. Most missing data occurred in the baseline EQ-5D-3L post-injury score. There were complete baseline, 6-week, and outcome (OMAS and TUG) data for 434/620 (70%) participants.

Predicting patient-reported functional outcome 6 months after ankle fracture

OMAS baseline model

TABLE 4 shows the eight baseline variables associated with 6-month OMAS and selected for the OMAS baseline model: pre-injury OMAS, alcohol consumed per week (units), post-injury EQ-5D-3L VAS, sex, walking distance, walking aid, smoking status, and health status. For instance, if all variables were kept constant, the 6-month OMAS for women was on average approximately 7 points lower than for equivalent men (p<0.001, 6-month OMAS median [IQR], 70 [50 - 80]). A 10-point

For peer review only

difference in the pre-injury OMAS for two identical individuals led to an average difference of approximately 2 points in the 6-month OMAS. The units of alcohol consumed in a week and the post-injury EQ-5D-3L VAS had marginal (p=0.043) and non-influential (p=0.102) effects on the 6-month OMAS, respectively. People who could walk further than 0.5 miles before injury had an approximately 8-point higher OMAS than those who could not, and people who required a walking aid before injury were more likely to have an approximately 8-point lower OMAS.

The initial model containing all candidate predictors had an R2 value of 0.25. The final model had an R2 value of 0.238 and an adjusted R2 of 0.223: the model's predictors explained approximately 22% of the outcome variation.

After bootstrapping, the optimism-corrected R2 performance estimate was 0.228. The model's performance on the internal validation was similar to that achieved on the original dataset, indicating no evidence for any overfitting.

OMAS baseline/6-week model

Adding the 6-week follow-up variables produced a model similar to the baseline model, minus post-injury EQ-5D-3L VAS, plus five 6-week follow-up variables: presence of radiological malalignment, range of motion in the injured ankle for dorsiflexion and plantar flexion, 6-week OMAS, and 6-week EQ-5D-3L (**TABLE 4**). A patient with radiological malalignment at 6 weeks would, on average, have a 6-month OMAS approximately 4 points lower than a patient without malalignment. Keeping all other variables constant, 20 degrees more ankle motion in both dorsiflexion and plantar flexion at 6 weeks would result in an approximately 4-point

For peer review only

higher 6-month OMAS. The higher the 6-week OMAS and EQ-5D-3L, the greater the 6-month OMAS ($p<0.001$ and $p=0.002$, respectively).

The baseline/6-week model had an adjusted R2 value of 0.261 which was higher than the baseline model's adjusted R2. Including the 6-week follow-up variables explained around 4% more variation than the baseline model.

After bootstrapping, the optimism-corrected R2 performance estimate was 0.264, similar to that on the original dataset. Approximately 26% of the variation in the 6-month OMAS was explained by the model, an increase of 3% from the baseline linear multivariable model.

In an exploratory analysis, a model using only the 6-week variables was developed. The adjusted R2 value was 0.123, which was significantly lower than the model performance for the baseline model.

Predicting objectively measured functional outcome 6 months after ankle fracture

TUG baseline model

TABLE 5 shows the baseline variables associated with the TUG time at 6 months and included in the baseline TUG model. On average women had around 6 seconds ($p<0.001$) longer TUG times than men (6-month TUG median [IQR], 18 seconds [14 – 23]). Holding all other variables constant, a 10-point increase in the mini-mental state examination score led to a 2 second decrease in the predicted TUG time

For peer review only

(p<0.001). A 10-year age difference between two otherwise identical individuals resulted in an 8-s longer predicted TUG time for the older individual (p<0.001). Those with higher post-injury EQ-5D-3L VAS were more likely to have a quicker TUG time at 6 months (p<0.001). People able to walk further than 0.5 mile before injury had a TUG time approximately 20 seconds quicker than an equivalent person who could only walk less than 0.5 mile (p<0.001). People who required a walking aid before injury were 19 s slower, on average, than those who did not. Fracture pattern and pre-injury OMAS predicted the TUG score well: a Weber C fracture pattern and low pre-injury OMAS predicted a longer TUG time.

The initial model containing all possible variables had an R2 value of 0.321, indicating that all of the variables chosen for consideration in model building together explained 32% of the variation in the TUG outcome. The final baseline model had an R2 value of 0.308 and an adjusted R2 of 0.298, indicating that the model's predictors explained approximately 30% of the outcome variation.

After bootstrapping, the optimism-corrected R2 performance estimate was 0.293. The model's performance on the internal validation was very similar to that achieved on the original dataset, explaining approximately 30% of the variation in the 6-month TUG score.

TUG baseline/6-week model

When the 6-week variables were included in building a model to predict the 6-month TUG time, four baseline variables were removed. The model-building process selected another baseline variable, home support, for inclusion in their place. Its

For peer review only

inclusion led to spurious results as there was little data in one of the subcategories (lives with carer/has external care support). The variable was therefore removed from the final model. The final model also included three 6-week variables: readmission, 6-week EQ-5D-3L, and 6-week EQ-5D-3L VAS (**TABLE 5**).

If a person was readmitted in the first 6 weeks after injury, they were predicted to have a 4 seconds faster TUG time at 6 months (p=0.04), if all other variables were held constant. Those with a better EQ-5D-3L score at 6 weeks were more likely to have a quicker TUG (p<0.001). If all other variables were kept constant, an increase of 20 points in the 6-week VAS resulted in a 1.5 seconds quicker time (p=0.04).

The baseline/6-week data model had a greater adjusted R2 (0.321) than the baseline data model (0.308). The model now explained 32% of the variability in TUG at 6 months, 2% more than that explained by the baseline data model.

After bootstrapping, optimism-corrected R2 performance estimate was 0.314. The model's performance on the internal validation dataset was very similar to that achieved on the original dataset, explaining approximately 31% of the variation in the 6-month TUG time.

In an exploratory analysis, a model using only the 6-week variables was developed. The adjusted R2 value was 0.109, which was lower than the model performance for the baseline model.

For peer review only

Sensitivity analysis

By using single imputation for the missing values in the 6-month OMAS and TUG outcomes, we assumed that the missing values occurred at random, so that variables in the dataset could predict the occurrence of missing values. To check how sensitive the four models were to this assumption, we conducted a complete-case analysis, which assumes that missing values occur completely at random.

Twenty-six participants were omitted from the OMAS models, leaving 592 participants. The same variables were selected for the baseline data model. The same variables were selected for the baseline/6-week data model, plus post-injury EQ5D-3L VAS and 'started partial weight bearing'. Both models had marginally lower adjusted R2 values: 0.205 for the baseline data model, 2% lower than the original model, and 0.256 for the baseline/6-week data model, 0.5% lower than the original model. After bootstrapping, the optimism-corrected R2 performance estimates of 0.215 for the baseline data model and 0.268 for the baseline/6-week data model.

Sixty-eight participants were omitted from the TUG models, leaving 550 participants. The baseline data model included the same variables as before, but replaced baseline post-injury EQ-5D-3L VAS with baseline recall pre-injury EQ-5D-3L VAS and omitted pre-injury OMAS. The baseline/6-week data model included the same variables as before, plus fracture pattern and walking aid. Both models had lower adjusted R2 values: 0.222 for the baseline data model, 8% lower than the original model, and 0.264 for the baseline/6-week data model, 6% lower than the original model. After bootstrapping, the optimism-corrected R2 performance estimates were 0.215 for the baseline data model and 0.253 for the baseline/6-week data model.

For peer review only

DISCUSSION

A prognostic model has the potential to inform anticipated recovery, and identify people who may benefit from additional monitoring or rehabilitation. We developed and internally validated four prognostic models for 6-month outcomes after ankle fracture using recommended methods[10] and a wide range of regularly collected data on plausible prognostic factors from hospital admission and at 6-week follow-up clinics. The prognostic models for the objectively measured mobility measure (TUG) explained about a third of the outcome variance, whereas the prognostic models for patient-reported ankle function (OMAS) explained about a quarter of the outcome variance. Adding 6-week follow-up variables to baseline variables provided only a modest benefit. This benefit arguably does not outweigh the logistical issues of obtaining patient information at 6 weeks to improve prediction accuracy. The models performed similarly on the development dataset and bootstrapped internal validation datasets.

The four models using commonly recorded clinical data have some ability to predict self-reported or objectively measured ankle function 6 months after unstable ankle fracture in adults aged over 60 years old. As there are limitations in predictive performance, we do not recommend using these prognostic models in clinical practice as decision-making tools in isolation. However, the models offer insights into prognosis after ankle fracture at the population level.

The variables of most predictive value across the prognostic models were sex, with females having worse outcomes than males; self-reported pre-injury walking distance, with those able to walk less than 0.5 miles doing worse than those able to

For peer review only

walk further; and pre-injury walking aid use, with those who did not need an aid faring better. A cohort of 584 severe ankle sprain participants also found that females had worse outcomes.[12] Shorter walking distances and use of a walking aid pre-injury are features of declining locomotor function, which is an important aspect of the domains assessed in the OMAS and TUG outcomes. They are likely to be related to a greater level of frailty pre-injury.[13]

The inferences presented here cannot be extended to propose causal relationships between the predictors and outcomes, as we did not consider issues such as confounding. As the aim and design of this study prioritised optimising prognostic accuracy, predictors were considered without considering whether they could form a causal pathway or not.[14]

Comparisons with other studies that have developed prognostic models for people after ankle fracture are challenging as, to the best of our knowledge, this is the first study to focus on older adults, use a larger cohort, and internally validate the developed models to adjust for optimism. A two-centre observational study of 60 adults (mean age 49) reported that ankle dorsiflexion and fracture classification (number of malleoli injured) predicted OMAS 6 months after cast removal (R^2 =0.4).[3] In a larger cohort of 150 adults (mean age 46), Lin and colleagues[7] developed a model to predict patient-reported lower limb function (Lower Extremity Functional Scale)[15] at 4 and 12 weeks after cast removal. The models were not internally validated, but were externally validated in a separate cohort of 94 participants. Eight predictors were examined. Pain and dorsiflexion after cast removal were included in the final model, which explained about 15% of the outcome variability in the

For peer review only

development stage and even less in the external validation.

The lack of predictive power of both our models and those in the literature indicate that functional outcomes after ankle fracture may be difficult to predict, or other prognostic factors may need to be considered to improve predictions of functional outcome. Our models may be missing informative predictors that were not captured in the AIM trial dataset. More detailed injury characteristics from specialist imaging could provide useful prognostic information. However, any techniques using specialist equipment or personnel would hamper clinical utility for routine use and would be difficult to implement if there were resource implications. Further research is recommended to investigate whether a wider range of psychosocial and environmental factors[16] can enhance predictive accuracy.

Similar results were found for the main and sensitivity analyses of both the OMAS and TUG models. The assumption that data were missing at random was therefore plausible and conducting single imputation before model building was probably appropriate.

This study was limited by the use of an existing clinical trial dataset for developing the prognostic model, as the choice of potential prognostic factors were restricted to those collected in the trial. Clinical trial cohorts are usually more selective than the wider clinical population. However, the AIM trial was a pragmatic study to enhance external validity. The use of a clinical trial cohort to develop prognostic models is also not uncommon, and has resulted in robust prognostic models for other ankle injury populations that have been successfully externally validated.[17] Strengths of

For peer review only

using this clinical trial dataset were that it had very low levels of missing data and that it reflected data that is or could be routinely collected during acute hospital admission and clinic follow-up.

CONCLUSION

We developed and internally validated prognostic models to predict functional outcomes 6 months after unstable ankle fracture in older adults using commonly recorded clinical data. These prognostic models have some ability to predict self-reported or objectively measured ankle function. Other potential predictors (e.g., psychosocial factors) should be investigated.

For peer review only

Author contributions

DJK devised the study, obtained and interpreted the data and led writing the manuscript. KV analysed and interpreted the data. KW obtained the data and contributed to the study design and interpretation. DM contributed to data management and analysis. MC contributed to the study design and data interpretation. GSC oversaw the analysis plan development and the conduct of the analysis. SEL obtained the data and contributed to the study design and interpretation. All authors critically reviewed the manuscript.

Acknowledgements

We thank the AIM trial participants and collaborators. We acknowledge Jennifer A. de Beyer of the Centre for Statistics in Medicine, University of Oxford, for English language editing and William Soanes for contributing to the statistical analysis plan.

Funding

This report is independent research supported by the National Institute for Health Research (NIHR Post Doctoral Fellowship, Dr David Keene, PDF-2016-09-056). The report was supported by the NIHR Biomedical Research Centre, Oxford. Professor Lamb receives funding from the NIHR Collaboration for Leadership in Applied Health Research and Care Oxford at Oxford Health NHS Foundation Trust.

Disclaimer

The views expressed in this publication are those of the author(s) and not necessarily those of the NHS, the National Institute for Health Research or the Department of Health.

For peer review only

Ethics approval

The National Research Ethics Service Oxfordshire Committee approved use of data. All participants gave written informed consent for data to be used.

Data sharing

All data requests should be submitted to the corresponding author for consideration. Access to anonymised data may be granted following review. Exclusive use will be retained until the publication of major outputs.

For peer review only

References

1. Kannus P, Niemi S, Parkkari J, et al. Declining incidence of fall-induced ankle fractures in elderly adults: Finnish statistics between 1970 and 2014. *Arch Orthop Trauma Surg* 2016;136(9):1243-6. doi: 10.1007/s00402-016-2524-7 [published Online First: 2016/07/23]
2. Beckenkamp PR, Lin CW, Chagpar S, et al. Prognosis of physical function following ankle fracture: a systematic review with meta-analysis. *J Orthop Sports Phys Ther* 2014;44(11):841-51, B2. doi: 10.2519/jospt.2014.5199
3. Hancock MJ, Herbert RD, Stewart M. Prediction of outcome after ankle fracture. *J Orthop Sports Phys Ther* 2005;35(12):786-92. [published Online First: 2006/07/20]
4. Keene D, James G, Lamb SE, et al. Factors associated with mobility outcomes in older people post-ankle fracture: An observational cohort study focussing on peripheral vessel function. *Injury* 2013;44(7):987-93. doi: http://dx.doi.org/10.1016/j.injury.2012.08.042
5. Lin CW, Moseley AM, Herbert RD, et al. Pain and dorsiflexion range of motion predict short- and medium-term activity limitation in people receiving physiotherapy intervention after ankle fracture: an observational study. *Aust J Physiother* 2009(1):31-7.
6. Keene DJ, Mistry D, Nam J, et al. The Ankle Injury Management (AIM) trial: a pragmatic, multicentre, equivalence randomised controlled trial and economic evaluation comparing close contact casting with open surgical reduction and internal fixation in the treatment of unstable ankle fractures in patients aged over 60 years. *Health Technol Assess* 2016;20(75):1-158. doi: 10.3310/hta20750 [published Online First: 2016/10/14]

For peer review only

7. Willett K, Keene DJ, Mistry D, et al. Close Contact Casting vs Surgery for Initial Treatment of Unstable Ankle Fractures in Older Adults: A Randomized Clinical Trial. *Jama* 2016;316(14):1455-63. doi: 10.1001/jama.2016.14719

8. Olerud C, Molander H. A scoring scale for symptom evaluation after ankle fracture. *Arch Orthop Trauma Surg* 1984;103(3):190-4. [published Online First: 1984/01/01]

9. Mathias S, Nayak US, Isaacs B. Balance in elderly patients: the "get-up and go" test. *Arch Phys Med Rehabil* 1986;67(6):387-9. [published Online First: 1986/06/01]

10. Steyerberg EW. Clinical prediction models : a practical approach to development, validation, and updating. New York: Springer 2009.

11. Collins GS, Reitsma JB, Altman DG, et al. Transparent Reporting of a multivariable prediction model for Individual Prognosis Or Diagnosis (TRIPOD). *Ann Intern Med* 2015;162(10):735-6. doi: 10.7326/L15-5093-2

12. Akacha KH, J.L. Lamb, S.E. Modelling treatment, age- and gender-specific recovery in acute injury studies. The University of Warwick Centre for Research in Statistical Methodology, 2010:1-12.

13. Middleton A, Fritz SL, Lusardi M. Walking speed: the functional vital sign. *J Aging Phys Act* 2015;23(2):314-22. doi: 10.1123/japa.2013-0236 [published Online First: 2014/05/09]

14. Tripepi G, Jager KJ, Dekker FW, et al. Testing for causality and prognosis: etiological and prognostic models. *Kidney Int* 2008;74(12):1512-15. doi: https://doi.org/10.1038/ki.2008.416

15. Binkley JM, Stratford PW, Lott SA, et al. The Lower Extremity Functional Scale (LEFS): scale development, measurement properties, and clinical application.

For peer review only

North American Orthopaedic Rehabilitation Research Network. *Phys Ther* 1999;79(4):371-83. [published Online First: 1999/04/14]

16. Kendrick D, Kelllezi B, Coupland C, et al. Psychological morbidity and health-related quality of life after injury: multicentre cohort study. *Qual Life Res* 2017;26(5):1233-50. doi: 10.1007/s11136-016-1439-7 [published Online First: 2016/10/28]

17. Keene DJ, Schlussel MM, Thompson J, et al. Prognostic models for identifying risk of poor outcome in people with acute ankle sprains: the SPRAINED development and external validation study. *Health Technol Assess* 2018;22(64):1-112. doi: 10.3310/hta22640 [published Online First: 2018/11/27]

For peer review only

TABLE 1: Pre-selected predictors at baseline.

	Measurement
**Baseline predictors**	
Age	Years
Sex	Male, female
Treatment received	Close contact cast/internal fixation surgery/other procedure
Fracture classification	Trans- infra-syndesmotic (Weber A and B) , supra-syndesmotic (Weber C)
Health status	Excellent, Very good, Good, Fair, Poor
Smoking status	Never, Ex-smoker, Yes
Admitted from	Own home, warden accommodation, acute hospital, community hospital, temporary residence
Home support	Lives alone, lives with someone, lives with carer, home care package
Alcohol status	Units per week
Cognitive function: Mini-Mental State Examination (MMSE)	Score (0-30, higher scores indicate better cognitive function)
Number of comorbidities	≥0
Walking distance pre-injury	About house, less than 100m, less than 0.5 mile, more than 0.5 mile
Walking aid pre-injury	None, one stick, two sticks, frame/rollator, wheelchair
Pre-injury ankle function: Pre-injury OMAS (recall of pre-injury status)	Score (0-100, higher score indicates better ankle function)
Health-related quality of life (HRQL): EQ-5D-3L score at: Day before injury Post-injury (at baseline assessment)	EQ-5D-3L score (upper bound equal to 1 indicates full health, 0 represents death, negative scores indicate a state worse than death)
EQ-5D-3L visual analogue scale (VAS) at: Day before injury Post-injury (at baseline assessment)	EQ-5D-3L VAS (0-100, higher scores indicate better HRQL)
**6-week follow-up predictors**	
OMAS at 6 weeks	Score (0-100)
EQ-5D-3L VAS 6 week	As baseline
EQ-5D-3L score at 6 weeks after injury	As baseline
Injured ankle range of dorsiflexion	Hand-held goniometry, degrees
Injured ankle range of plantar flexion	Hand-held goniometry, degrees
Readmission to hospital	Yes/No
Started partial weight bearing by 6 weeks	Yes/No
Radiological malalignment in 6 week radiograph	Yes/No

*radiological malalignment at 6 weeks was assessed by a combination of bespoke measurement software and verification by two experienced consultant surgeon using the criteria: radiograph demonstrating any one or combination of showing talar subluxation > 2 mm [talar shift], excessive talar tilt [> 2°], or a diastasis [tibiofibular clear space ≥ 5 mm].
OMAS: Olerud and Molander Ankle Score

For peer review only

TABLE 2: Median [IQR] values for the baseline and 6-week continuous variables and the 6 month outcomes.

Continuous variable	n	Median [IQR]
6-month OMAS score	592	70 [50 - 80]
6-month TUG	550	18 [14 – 23]
Age (years)	618	70 [65 - 76]
Mini Mental State Examination (MMSE)	556	29 [27 - 30]
Number of alcohol units consumed in a week	614	2 [0 - 10]
EQ-5D-3L VAS	557	85 [75 - 95]
EQ-5D-3L VAS post-injury	521	58 [40 - 75]
Number of comorbidities	618	1 [1 - 2]
Pre-injury OMAS	618	100 [80 - 100]
EQ-5D-3L score	557	1 [0.80 - 1]
EQ-5D-3L score post-injury	522	0.02 [-0.06 – 0.16]
Injured ankle range dorsiflexion	578	5 [0 - 10]
Injured ankle range plantar flexion	578	20 [12 - 30]
6-week OMAS	605	35 [30 - 50]
6-week EQ-5D-3L score	550	0.52 [0.31 – 0.71]
6-week EQ-5D-3L VAS	550	75 [60 - 86]

TUG: Timed Up and Go; OMAS: Olerud and Molander Ankle Score

For peer review only

Table 3: OMAS and TUG median [IQR] for baseline and 6-week categorical variables.

Categorical variable	n	Median OMAS [IQR]	n	Median TUG [IQR]
Sex				
Male	151	75 [60 – 90]	142	17 [14 – 19]
Female	441	65 [48 – 80]	408	18 [15 – 24]
Smoking status				
Current smoker	55	65 [50 – 80]	51	18 [14 – 23]
Ex-smoker	234	68 [50 – 80]	216	18 [14 – 23]
Never	303	70 [50 – 80]	283	18 [14 – 23]
Health status				
Excellent	135	75 [60 – 85]	132	15 [13 – 19]
Very good	250	70 [55 – 85]	233	18 [14 – 21]
Good	153	60 [45 – 80]	142	20 [16 – 27]
Fair	50	53 [35 – 70]	41	24 [18 – 33]
Poor	4	48 [11 – 88]	2	44 [28 – 59]
Admitted from				
Own home	576	70 [50 – 80]	539	18 [14 – 23]
Warden accommodation	6	68 [41 – 86]	4	18 [17 – 25]
Acute hospital	2	55 [40 – 70]	2	29 [18 – 40]
Community hospital	2	75 [70 – 80]	1	14 [14 – 14]
Temporary residence	6	75 [59 – 91]	4	14 [12 – 20]
Home support				
Lives alone	188	70 [50 – 85]	173	19 [15 – 24]
Lives with someone	398	70 [50 – 80]	375	17 [14 – 22]
Lives with carer	1	35 [35 – 35]	1	34 [34 – 34]
Home care package	5	40 [25 – 55]	1	15 [15 – 15]
Walking aid pre-injury				
None	512	70 [55 – 85]	491	17 [14 – 21]
One stick	58	45 [35 – 61]	50	26 [21 – 32]
Two sticks	6	48 [30 – 65]	4	37 [18 – 59]
Frame/Rollator	13	40 [25 – 70]	4	88 [40 – 192]
Wheelchair	3	70 [45 – 95]	1	46 [46 – 46]
Walking distance pre-injury				
About house	17	55 [35 – 70]	6	29 [26 – 112]
Less than 100m	33	40 [28 – 58]	28	37 [27 – 47]
Less than 0.5 mile	58	55 [40 – 75]	52	22 [17 – 28]
More than 0.5 mile	484	70 [55 – 85]	464	17 [14 – 21]
Fracture pattern				
Weber A and B	516	70 [50 – 80]	480	18 [14 – 23]
Weber C	76	65 [45 – 79]	70	18 [15 – 23]
Treatment received				
Close contact casting	271	70 [50 – 80]	246	18 [15 – 23]
Internal fixation surgery	308	70 [55 – 80]	292	17 [14 – 22]
Other	13	55 [38 – 85]	12	20 [17 – 29]
Readmission				
No	542	70 [50 – 80]	506	18 [14 – 23]
Yes	50	63 [50 – 75]	44	18 [14 – 21]
Partial weight date present				
No	224	70 [50 – 85]	213	17 [14 – 22]
Yes	368	70 [50 – 80]	337	18 [14 – 23]

For peer review only

Radiological malalignment				
No	365	70 [50 – 80]	343	18 [14 – 23]
Yes	227	65 [45 – 80]	207	18 [15 – 23]

For peer review only

TABLE 4: Univariable analysis and the final baseline and baseline plus 6-week follow-up linear multivariable models for the 6-month OMAS score

Variable	Univariable analysis		Final multivariable baseline model		Final multivariable baseline plus 6 week follow-up model	
	β coefficient [95% CI]	p-value	β coefficient [95% CI]	p-value	β coefficient [95% CI]	p-value
Baseline						
Constant			44.34 [30.04, 58.64]	<0.001	34.62 [20.00, 49.23]	<0.001
Pre-injury OMAS score	0.48 [0.39, 0.57]	<0.001	0.22 [0.09, 0.34]	0.001	0.19 [0.07, 0.31]	0.002
Alcohol consumed per week (units)	0.34 [0.16, 0.52]	<0.001	0.14 [-0.03, 0.32]	0.102	0.23 [0.07, 0.40]	0.007
EQ-5D-3L VAS Score (post-injury)	0.18 [0.10, 0.25]	<0.001	0.08 [0.003, 0.15]	0.043		
Sex		<0.001		<0.001		0.010
Female	-8.94 [-12.82, -5.05]		-6.90 [-10.75, -3.06]		-4.99 [-8.79, -1.19]	
Walking distance*		<0.001		0.002		0.015
>0.5mile	19.10 [15.01, 23.19]		7.79 [2.97, 12.61]		5.90 [1.16, 10.64]	
Walking aid		<0.001		0.003		0.006
Yes	-20.31 [-24.95, -15.66]		-8.35 [-13.89, -2.82]		-7.60 [13.01, -2.20]	
Smoking status†		0.185		0.049		0.061
Ex-smoker	-1.57 [-7.87, 4.72]		-3.31 [-8.93, 2.30]		-3.26 [-8.73, 2.20]	
Never	1.83 [-4.33, 8.00]		0.85 [-4.69, 6.38]		0.62 [-4.76, 6.00]	
Health status‡		<0.001		0.023		0.050
Very good	-4.19 [-8.50, 0.13]		-1.79 [-5.82, 2.24]		-2.25 [-6.17, 1.66]	
Good	-13.42 [-18.13, -8.70]		-6.90, [-11.48, -2.32]		-6.50 [-10.93, -2.07]	
Fair	-20.69 [-27.41, -13.97]		-2.97 [-10.32, 4.38]		-4.48 [-11.51, 2.56]	
Poor	-23.48 [-44.30, -2.66]		5.94 [-14.25, 26.14]		2.95 [-16.61, 22.51]	
Patient age	-0.54 [-0.77, -0.31]	<0.001				
MMSE	1.21 [0.43, 2.00]	0.003				
EQ-5D-3L VAS Score (day before injury)	0.35 [0.25, 0.46]	<0.001				
EQ-5D-3L score (day before injury)	32.3 [23.07, 41.53]	<0.001				
EQ-5D-3L score (post-injury)	11.24 [4.65, 17.83]	<0.001				
Number of comorbidities	-3.58 [-4.89, -2.28]	<0.001				
Fracture pattern		0.175				
Weber C	-3.59 [-8.78, 1.60]					

For peer review only

Admitted from§		0.441				
Warden accommodation	-8.31 [-23.57, 6.96]					
Acute hospital	-10.01 [-40.38, 20.37]					
Community hospital	9.99 [-20.38, 40.37]					
Temporary residence	10.96 [-5.35, 27.26]					
Treatment received‖		0.249				
ORIF	1.67 [-1.83, 5.18]					
Other	-6.33 [-16.75, 4.09]					
Home support ¶		0.003				
Live alone or with someone	26.11 [8.63, 43.59]					
Additonal 6 week variables						
6-week EQ-5D-3L VAS	0.32 [0.21, 0.42]	<0.001				
Readmission to hospital						
Yes	-3.30 [-9.49, 2.89]	0.295				
Started partial weight bearing		0.575				
Yes	-1.01 [-4.54, 2.52]					
Radiological malalignment present		0.124				0.025
Yes	-2.78 [-6.33, 0.77]				-3.54 [-6.62, -0.45]	
Range of injured ankle motion Dorsiflexion	0.15 [-0.04, 0.34]	0.131			0.18 [0.007, 0.35]	0.041
Range of injured ankle motion Plantar flexion	0.14 [0.01, 0.28]	0.032			0.17 [0.05, 0.28]	0.006
6-week OMAS	0.33 [0.22, 0.44]	<0.001			0.19 [0.09, 0.30]	<0.001
6-week EQ-5D-3L score	25.69 [18.66, 32.72]	<0.001			10.21 [3.64, 16.78]	0.002

*Reference category for walking distance is “<0.5 mile”
†Reference category for smoking status is “Current smoker”
‡Reference category for health status is “Excellent”
§Reference category for admitted from is “Own home”
‖Reference category for treatment received from is “CCC”
¶Reference category for home support is “live with care or has external care support”
NB: EQ-5D-3L VAS Score (post-injury) omitted from final model by backward selection and correlation with 6-week score.
OMAS: Olerud and Molander Ankle Scale; MMSE: Mini-Mental State Examination; ORIF: Open Reduction and Internal Fixation; VAS: Visual Analogue Scale

For peer review only

TABLE 5: Univariable analysis and the final baseline and baseline plus 6-week follow-up linear multivariable models for the 6-month TUG score

Variable	Univariable analysis		Final multivariable baseline model		Final multivariable baseline plus 6 week follow-up model	
	β coefficient [95% CI]	p-value	β coefficient [95% CI]	p-value	β coefficient [95% CI]	p-value
Baseline						
Constant			24.86 [-0.31, 50.03]	0.05	51.72 [29.34, 74.11]	<0.001
Pre-injury OMAS score	-0.38 [-0.45, -0.30]	<0.001	-0.09 [-0.19, 0.0007]	0.05		
Alcohol consumed per week (units)	-0.24 [-0.38, -0.10]	0.001				
EQ-5D-3L VAS Score (post-injury)	-0.12 [-0.18, -0.06]	<0.001	-0.05 [-0.10, 0.006]	0.08		
Sex		<0.001		0.008		0.02
Female	5.64 [2.51, 8.78]		3.61 [0.93, 6.29]		3.30 [0.57, 6.03]	
Walking distance*		<0.001		<0.001		<0.001
>0.5mile	-19.67 [-22.80, -16.53]		-12.26 [-15.87, -8.65]		-9.90 [-13.30, -6.50]	
Walking aid		<0.001		0.001		<0.001
Yes	19.00 [15.35, 22.64]		7.67 [3.34, 11.99]		6.10 [2.35, 9.85]	
Smoking status†		0.33				0.04
Ex-smoker	-0.06 [-5.11, 4.99]				-1.26 [-5.48, 2.96]	
Never	-2.15 [-7.10, 2.81]				-3.93 [-8.07, 0.21]	
Health status‡		<0.001				
Very good	1.62 [-1.84, 5.07]					
Good	7.68 [3.90, 11.46]					
Fair	18.08 [12.70, 23.47]					
Poor	23.27 [6.60, 39.94]					
Patient age	0.79 [0.61, 0.97]	<0.001	0.36 [0.18, 0.54]	<0.001	0.37 [0.20, 0.54]	<0.001
MMSE	-2.15 [-2.75, -1.54]	<0.001	-0.89 [-1.46, -0.32]	0.002	-1.35 [-1.91, -0.79]	<0.001
EQ-5D-3L VAS Score (day before injury)	-0.35 [-0.43, -0.26]	<0.001				
EQ-5D-3L score (day before injury)	-21.22 [-28.46, -13.97]	<0.001	6.73 [-0.99, 14.44]	0.09		
EQ-5D-3L score (post-injury)	-8.48 [-13.92, -3.03]	0.002				
Number of comorbidities	3.43 [2.34, 4.46]	<0.001				
Fracture pattern		0.03		0.03		
Weber C	4.61 [0.46, 8.76]		3.97 [0.44, 7.50]			
Admitted from§		0.67				
Warden accommodation	0.82 [-11.44, 13.07]					
Acute hospital	6.72 [-17.66, 31.11]					

Community hospital	-0.50 [-24.89, 23.89]					
Temporary residence	-9.53 [-22.62, 3.56]					
Treatment received‖		0.35				
ORIF	-0.94 [-3.75, 1.87]					
Other	4.95 [-3.41, 13.31]					
Home support¶		0.03				
Live alone or with someone	-15.40 [-29.47, -1.34]					
Additonal 6 week variables						
6-week EQ-5D-3L VAS	-0.24 [-0.32, -0.16]	<0.001			-0.07 [-0.14, 0.00]	0.05
Readmission to hospital		0.06				0.03
Yes	-4.83 [-9.81, 0.15]				-4.49 [-8.60, -0.38]	
Started partial weight bearing		0.96				
Yes	0.08 [-2.77, 2.92]					
Radiological malalignment present		0.24				
Yes	-1.73 [-4.59, 1.13]					
Range of injured ankle motion Dorsiflexion	-0.005 [-0.16, 0.15]	0.95				
Range of injured ankle motion Plantar flexion	0.01 [-0.09, 0.12]	0.83				
6-week OMAS	-0.13 [-0.22, -0.04]	0.005				
6-week EQ-5D-3L score	-23.76 [-29.46, -18.06]	<0.001			-11.11 [-16.13, -6.09]	<0.001

*Reference category for walking distance is "<0.5 mile"
†Reference category for smoking status is "Current smoker"
‡Reference category for health status is "Excellent"
§Reference category for admitted from is "Own home"
‖Reference category for treatment received from is "CCC"
¶Reference category for home support is "live with care or has external care support"
NB: EQ-5D-3L VAS Score (post-injury) omitted from final model by backward selection and correlation with 6-week score.
OMAS: Olerud and Molander Ankle Scale; MMSE: Mini-Mental State Examination; ORIF: Open Reduction and Internal Fixation; VAS: Visual Analogue Scale

For peer review only

TRIPOD Checklist: Prediction Model Development and Validation

Section/Topic	Item		Checklist Item	Page
**Title and abstract**				
Title	1	D;V	Identify the study as developing and/or validating a multivariable prediction model, the target population, and the outcome to be predicted.	Title page
Abstract	2	D;V	Provide a summary of objectives, study design, setting, participants, sample size, predictors, outcome, statistical analysis, results, and conclusions.	Abstract page
**Introduction**				
Background and objectives	3a	D;V	Explain the medical context (including whether diagnostic or prognostic) and rationale for developing or validating the multivariable prediction model, including references to existing models.	Intro section
	3b	D;V	Specify the objectives, including whether the study describes the development or validation of the model or both.	Intro section, Objectives subsetion
**Methods**				
Source of data	4a	D;V	Describe the study design or source of data (e.g., randomized trial, cohort, or registry data), separately for the development and validation data sets, if applicable.	Study design subsection
	4b	D;V	Specify the key study dates, including start of accrual; end of accrual; and, if applicable, end of follow-up.	Study design subsection
Participants	5a	D;V	Specify key elements of the study setting (e.g., primary care, secondary care, general population) including number and location of centres.	Study design subsection
	5b	D;V	Describe eligibility criteria for participants.	Participants subsection
	5c	D;V	Give details of treatments received, if relevant.	Participants subsection
Outcome	6a	D;V	Clearly define the outcome that is predicted by the prediction model, including how and when assessed.	Outcome measures subsection
	6b	D;V	Report any actions to blind assessment of the outcome to be predicted.	Outcome measures, secondary outcomes section
Predictors	7a	D;V	Clearly define all predictors used in developing or validating the multivariable prediction model, including how and when they were measured.	Predictors subsection; Table 1
	7b	D;V	Report any actions to blind assessment of predictors for the outcome and other predictors.	Predictors subsection
Sample size	8	D;V	Explain how the study size was arrived at.	Statistical analysis subsection, first sentence
Missing data	9	D;V	Describe how missing data were handled (e.g., complete-case analysis, single imputation, multiple imputation) with details of any imputation method.	Statistical analysis subsection, end of first paragraph
Statistical analysis methods	10a	D	Describe how predictors were handled in the analyses.	Stats section, paragraph three.
	10b	D	Specify type of model, all model-building procedures (including any predictor selection), and method for internal validation.	Stats section, paragraph two.
	10c	V	For validation, describe how the predictions were calculated.	Stats section, paragraph five
	10d	D;V	Specify all measures used to assess model performance and, if relevant, to compare multiple models.	Stats section, end of paragraph four
	10e	V	Describe any model updating (e.g., recalibration) arising from the validation, if done.	Stats section, paragraph five
Risk groups	11	D;V	Provide details on how risk groups were created, if done.	N/A
Development vs. validation	12	V	For validation, identify any differences from the development data in setting, eligibility criteria, outcome, and predictors.	Internal validation
**Results**				
Participants	13a	D;V	Describe the flow of participants through the study, including the number of participants with and without the outcome and, if applicable, a summary of the follow-up time. A diagram may be helpful.	Participants subsection, Tables 2 and 3
	13b	D;V	Describe the characteristics of the participants (basic demographics, clinical features, available predictors), including the number of participants with missing data for predictors and outcome.	Tables 2 and 3
	13c	V	For validation, show a comparison with the development data of the distribution of important variables (demographics, predictors and outcome).	Internal validation
Model development	14a	D	Specify the number of participants and outcome events in each analysis.	Tables 4 and 5
	14b	D	If done, report the unadjusted association between each candidate predictor and outcome.	Tables 4 and 5

For peer review only

TRIPOD Checklist: Prediction Model Development and Validation

Model specification	15a	D	Present the full prediction model to allow predictions for individuals (i.e., all regression coefficients, and model intercept or baseline survival at a given time point).	Tables 4 and 5
	15b	D	Explain how to the use the prediction model.	Tables 4 and 5 contain data to calculate model
Model performance	16	D;V	Report performance measures (with CIs) for the prediction model.	Tables 2 and 5 and in text for each model
Model-updating	17	V	If done, report the results from any model updating (i.e., model specification, model performance).	In text for each model
**Discussion**				
Limitations	18	D;V	Discuss any limitations of the study (such as nonrepresentative sample, few events per predictor, missing data).	Discussion, paragraph eight
Interpretation	19a	V	For validation, discuss the results with reference to performance in the development data, and any other validation data.	Internal validation
	19b	D;V	Give an overall interpretation of the results, considering objectives, limitations, results from similar studies, and other relevant evidence.	Discussion, paragraphs one to seven
Implications	20	D;V	Discuss the potential clinical use of the model and implications for future research.	Discussion, paragraph two
**Other information**				
Supplementary information	21	D;V	Provide information about the availability of supplementary resources, such as study protocol, Web calculator, and data sets.	N/A
Funding	22	D;V	Give the source of funding and the role of the funders for the present study.	Funding statement section

*Items relevant only to the development of a prediction model are denoted by D, items relating solely to a validation of a prediction model are denoted by V, and items relating to both are denoted D;V. We recommend using the TRIPOD Checklist in conjunction with the TRIPOD Explanation and Elaboration document.

For peer review only

BMJ Open

Predicting Patient-reported and Objectively Measured Functional Outcome Six Months after Ankle Fracture in People Aged 60 Years or Over in the United Kingdom: Prognostic Model Development and Internal Validation

Journal:	*BMJ Open*
Manuscript ID	bmjopen-2019-029813.R1
Article Type:	Research
Date Submitted by the Author:	18-Apr-2019

Complete List of Authors:	Keene, David; University of Oxford Nuffield Department of Orthopaedics Rheumatology and Musculoskeletal Sciences,<br>Vadher, Karan; University of Oxford Nuffield Department of Orthopaedics Rheumatology and Musculoskeletal Sciences<br>Willett, Keith; University of Oxford, Nuffield Department of Orthopaedics, Rheumatology and Musculoskeletal Sciences<br>Mistry, Dipesh; University of Warwick, Warwick Clinical Trial Unit<br>Costa, Matthew; University of Oxford, Nuffield Department of Orthopaedics, Rheumatology, and Musculoskeletal Sciences<br>Collins, Gary; University of Oxford, Centre for Statistics in Medicine<br>Lamb, Sarah; University of Oxford, Ndorms

**Primary Subject Heading**:	Surgery
Secondary Subject Heading:	Rehabilitation medicine
Keywords:	ankle fractures, ankle injuries, osteoporotic fractures, prognosis

SCHOLARONE™
Manuscripts

Predicting Patient-reported and Objectively Measured Functional Outcome Six Months after Ankle Fracture in People Aged 60 Years or Over in the United Kingdom: Prognostic Model Development and Internal Validation

David J. Keene, D.Phil[1,2]

Karan Vadher, MSc[1]

Keith Willett, FRCS, MBBS[1]

Dipesh Mistry, PhD[3]

Matthew Costa, FRCS, PhD[1,2]

Gary S. Collins, PhD[1]

Sarah E. Lamb, D.Phil[1,2]

[1]Nuffield Department of Orthopaedics, Rheumatology and Musculoskeletal Sciences, University of Oxford, Oxford, UK.

[2]Oxford NIHR Biomedical Research Centre, Oxford University Hospitals NHS Foundation Trust, UK.

[3]Warwick Clinical Trials Unit, University of Warwick, Coventry, UK.

Address correspondence to David Keene, Kadoorie Research Centre, John Radcliffe Hospital, Oxford, OX3 9DU, UK

E-mail: david.keene@ndorms.ox.ac.uk

Word count: 3550

For peer review only

Abstract

Objective: To predict functional outcomes 6 months after ankle fracture in people aged ≥60 years using post-treatment and 6-week follow-up data to inform anticipated recovery, and identify people who may benefit from additional monitoring or rehabilitation.

Design: Prognostic model development and internal validation.

Setting: 24 National Health Service hospitals, UK.

Methods: Participants were the Ankle Injury Management clinical trial cohort (n=618) (ISRCTN04180738), aged 60–96 years, 459/618 (74%) female, treated surgically or conservatively for unstable ankle fracture.

Predictors were injury and socio-demographic variables collected at baseline (acute hospital setting) and 6-week follow-up (clinic).

Outcome measures were 6-month post-injury (primary) self-reported ankle function, using the Olerud and Molander Ankle Score (OMAS), and (secondary) Timed Up and Go (TUG) test by blinded assessor. Missing data was managed with Single imputation. Multivariable linear regression models were built to predict OMAS or TUG, using baseline variables or baseline and 6-week follow-up variables. Models were internally validated using bootstrapping.

Results: The OMAS baseline data model included: alcohol per week (units), post-injury EQ-5D-3L visual analogue scale (VAS), sex, pre-injury walking distance and walking aid use, smoking status, and perceived health status. The baseline/6-week data model included the same baseline variables, minus EQ-5D-3L VAS, plus five 6-week predictors: radiological malalignment, injured ankle dorsiflexion and plantarflexion range of motion, and 6-week OMAS and EQ-5D-3L. The models explained approximately 23% and 26% of the outcome variation, respectively.

For peer review only

Similar baseline and baseline/6-week data models to predict TUG explained around 30% and 32% of the outcome variation, respectively.

Conclusions: Predictive accuracy of the prognostic models using commonly recorded clinical data to predict self-reported or objectively measured ankle function was relatively low and therefore unlikely to be beneficial for clinical practice and counselling of patients. Other potential predictors (e.g., psychological factors such as catastrophising and fear avoidance) should be investigated to improve predictive accuracy.

Key words: ankle fractures, ankle injuries, osteoporotic fractures, prognosis

For peer review only

Strengths and limitations of this study

- Developed and internally validated prognostic models using robust methods.
- The AIM trial was a pragmatic study to enhance generalisability and had very low levels of missing data and is reflective of data that is or could be routinely collected during acute hospital admission and clinic follow-up.
- Use of an existing clinical trial dataset for developing the prognostic model restricted the choice of potential prognostic factors to those collected in the trial.

For peer review only

INTRODUCTION

Background

Ankle fractures in older people are increasing in number as the population ages.[1] Older people have a worse prognosis than younger adults for recovering function after fractures.[2] However, there is limited evidence on which injury, treatment, and socio-demographic factors predict functional outcomes after ankle fracture in older adults. Previous studies have used small cohorts and few predictive factors.[3-5]

Rationale

A multicentre randomised clinical trial of close contact casting versus surgery included 620 people aged 60 years and over with an unstable ankle fracture and found equivalence in outcomes between the treatment groups.[6 7] Irrespective of the initial fracture treatment, considerable outcome variation was evident in the trial cohort, highlighting the value of investigating which combination of socio-demographic and clinical prognostic factors could predict functional outcomes in this patient group. A prognostic model could inform patient counselling about prognosis and identify people who might benefit from additional monitoring or rehabilitation.

Objectives

Develop and internally validate a prognostic model to predict (1) patient-reported and (2) objectively measured functional outcome 6 months after ankle fracture in people aged 60 years or over using socio-demographic and clinical data collected in the acute phase and at 6-week follow-up.

For peer review only

METHODS

Study design and setting

Design Data from the Ankle Injury Management (AIM) trial cohort (registration: ISRCTN04180738) were used to develop and internally validate prognostic models. AIM was a pragmatic, multicentre, randomised equivalence trial and economic evaluation comparing close contact casting with open surgical reduction and internal fixation in the treatment of unstable ankle fractures in people aged over 60 years. The trial methods and results have been published elsewhere.[6 7] Participants were recruited from May 2004 in a pilot centre, then from 24 centres (general hospitals and major trauma centres) in the UK from July 2010 to November 2013. The primary endpoint was 6-month follow-up. All participants gave written informed consent for data to be used.

Participants

Eligible participants were aged 60 years or over and had an acute, overtly unstable ankle fracture (displaced or clinically unstable). Participants with insulin-dependent diabetes mellitus, active leg ulceration, critical limb ischaemia, open fracture, serious concomitant disease, substantial cognitive impairment, or ankle arthritis, or who were not fit for anaesthesia, were excluded. Participants were allocated to usual care (open reduction and internal fixation surgery) or a minimally padded close contact cast. Both treatments were conducted under anaesthesia by an orthopaedic surgeon.

Outcome measures

Primary outcome: The primary outcome to be predicted was ankle function,

For peer review only

measured using the Olerud and Molander Ankle Score (OMAS)[8] 6 months after fracture. The OMAS is a widely used questionnaire to assess outcomes after ankle fracture, covering a range of symptoms and mobility limitations. It is measured on a 0 to 100 scale, with lower scores representing worse ankle function. Participants reported their OMAS at follow-up clinics or, if unable to attend, over the telephone. If the participant did not have sufficient dexterity to complete the questionnaire independently the researcher acted as scribe.

Secondary outcome: The secondary outcome to be predicted was the Timed Up and Go (TUG) test,[9] an objective measurement of mobility conducted by a blinded outcome assessor. Participants were timed standing up from a chair, walking 8.6 m, turning, and returning to sit in the start position. Participants' ankles had a dressing applied to obscure the presence of lack of surgical incision scars. The TUG test is responsive, valid and reliable assessment of mobility in older adults that has been shown to be predictive of falls risk and functional decline.[10-12]

Predictors

Predictor variables were selected based on clinical rationale and availability in the clinical trial dataset (**TABLE 1**). Two models were produced for each outcome to investigate whether predictor variables collected at 6-week follow-up clinics improved the baseline prediction. Predictor measurements were obtained from participant questionnaires or clinical assessments. Two experienced orthopaedic surgeons with no access to the clinical data assessed fracture misalignment at 6 weeks on anteroposterior or mortise and lateral radiographs.

For peer review only

Statistical analysis

Sample size was constrained to the size of the AIM trial cohort as this was a pre-existing dataset. The number of variables was limited to those plausibly related the outcome. The OMAS and TUG multivariable models were built using data from 620 participants. We initially assessed the covariates by plotting scatter graphs of outcomes and each continuous covariate and comparing each covariate with another. If highly correlated predictors were found, only one was included in the multivariable modelling. Any predictors or participants with >90% missing data were excluded. Remaining missing data were dealt with using single imputation before building the model.

As the outcomes were continuous, the prognostic models were developed under a linear regression modelling framework. The predictors (**TABLE 1**) were included as independent variables in the model. Backwards elimination was performed to derive the models using the Akaike information criterion (AIC).

For peer review only

TABLE 1: Pre-selected predictors at baseline.

	Measurement
**Baseline predictors**	
Age	Years
Sex	Male, female
Treatment received	Close contact cast/internal fixation surgery/other procedure
Fracture classification	Trans- infra-syndesmotic (Weber A and B) , supra-syndesmotic (Weber C)
Health status	Excellent, Very good, Good, Fair, Poor
Smoking status	Never, Ex-smoker, Yes
Admitted from	Own home, warden accommodation, acute hospital, community hospital, temporary residence
Home support	Lives alone, lives with someone, lives with carer, home care package
Alcohol status	Units per week
Cognitive function:<br>Mini-Mental State Examination (MMSE)	Score (0-30, higher scores indicate better cognitive function)
Number of comorbidities	≥0
Walking distance pre-injury	About house, less than 100m, less than 0.5 mile, more than 0.5 mile
Walking aid pre-injury	None, one stick, two sticks, frame/rollator, wheelchair
Pre-injury ankle function:<br>Pre-injury OMAS (recall of pre-injury status)	Score (0-100, higher score indicates better ankle function)
Health-related quality of life (HRQL):<br>EQ-5D-3L score at:<br>Day before injury<br>Post-injury (at baseline assessment)	EQ-5D-3L score (upper bound equal to 1 indicates full health, 0 represents death, negative scores indicate a state worse than death)
EQ-5D-3L visual analogue scale (VAS) at:<br>Day before injury<br>Post-injury (at baseline assessment)	EQ-5D-3L VAS (0-100, higher scores indicate better HRQL)
**6-week follow-up predictors**	
OMAS at 6 weeks	Score (0-100)
EQ-5D-3L VAS 6 week	As baseline
EQ-5D-3L score at 6 weeks after injury	As baseline
Injured ankle range of dorsiflexion	Hand-held goniometry, degrees
Injured ankle range of plantar flexion	Hand-held goniometry, degrees
Readmission to hospital	Yes/No
Started partial weight bearing by 6 weeks	Yes/No
Radiological malalignment in 6 week radiograph	Yes/No

*radiological malalignment at 6 weeks was assessed by a combination of bespoke measurement software and verification by two experienced consultant surgeon using the criteria: radiograph demonstrating any one or combination of showing talar subluxation > 2 mm [talar shift], excessive talar tilt [> 2°], or a diastasis [tibiofibular clear space ≥ 5 mm].
OMAS: Olerud and Molander Ankle Score

For peer review only

Categorical variables were collapsed into binary variables to ensure sufficient data points in each subcategory. The use of an assistive device for walking before injury was collapsed into whether the patient used assistance (yes: one stick, two sticks, frame/rollator, or wheelchair) or not (no). Walking distance before injury was split into <0.5 miles and >0.5 miles, reflecting the person's walking endurance. Home support was split into lives alone/with someone or lives with carer/has external care support. Continuous variables were investigated for nonlinearity with the outcome using multivariable fractional polynomials.

Clinically plausible interactions were examined for inclusion in the model. Model performance was assessed by calculating the adjusted R^2.

The prognostic models were internally validated through bootstrapping using 200 bootstrap samples (replaying all variable selection procedures) to correct for optimism and quantify and adjust the model for overfitting.[13] All analyses were conducted using Stata 14.2. We followed the TRIPOD statement when reporting this study.[14]

Patient and Public Involvement

Patient and public involvement (PPI) representatives provided feedback on the research proposal in the planning stages of this study. A PPI representative was an independent member of the AIM trial steering committee.

For peer review only

RESULTS

Participants

The dataset contained 620 participants. Participants were aged median 70 (IQR 65 – 76) years and 459/618 (74%) were female. **TABLES 2 and 3** list the participant characteristics considered for building the models, the median [IQR] values for continuous variables, and the median OMAS and TUG values for categorical variables.

TABLE 2: Median [IQR] values for the baseline and 6-week continuous variables and the 6 month outcomes.

Continuous variable	n	Median [IQR]
6-month OMAS score	592	70 [50 - 80]
6-month TUG	550	18 [14 – 23]
Age (years)	618	70 [65 - 76]
Mini Mental State Examination (MMSE)	556	29 [27 - 30]
Number of alcohol units consumed in a week	614	2 [0 - 10]
EQ-5D-3L VAS	557	85 [75 - 95]
EQ-5D-3L VAS post-injury	521	58 [40 - 75]
Number of comorbidities	618	1 [1 - 2]
Pre-injury OMAS	618	100 [80 - 100]
EQ-5D-3L score	557	1 [0.80 - 1]
EQ-5D-3L score post-injury	522	0.02 [-0.06 – 0.16]
Injured ankle range dorsiflexion	578	5 [0 - 10]
Injured ankle range plantar flexion	578	20 [12 - 30]
6-week OMAS	605	35 [30 - 50]
6-week EQ-5D-3L score	550	0.52 [0.31 – 0.71]
6-week EQ-5D-3L VAS	550	75 [60 - 86]

TUG: Timed Up and Go; OMAS: Olerud and Molander Ankle Score

For peer review only

Table 3: OMAS and TUG median [IQR] for baseline and 6-week categorical variables.

Categorical variable	n	Median OMAS [IQR]	n	Median TUG [IQR]
Sex				
Male	151	75 [60 – 90]	142	17 [14 – 19]
Female	441	65 [48 – 80]	408	18 [15 – 24]
Smoking status				
Current smoker	55	65 [50 – 80]	51	18 [14 – 23]
Ex-smoker	234	68 [50 – 80]	216	18 [14 – 23]
Never	303	70 [50 – 80]	283	18 [14 – 23]
Health status				
Excellent	135	75 [60 – 85]	132	15 [13 – 19]
Very good	250	70 [55 – 85]	233	18 [14 – 21]
Good	153	60 [45 – 80]	142	20 [16 – 27]
Fair	50	53 [35 – 70]	41	24 [18 – 33]
Poor	4	48 [11 – 88]	2	44 [28 – 59]
Admitted from				
Own home	576	70 [50 – 80]	539	18 [14 – 23]
Warden accommodation	6	68 [41 – 86]	4	18 [17 – 25]
Acute hospital	2	55 [40 – 70]	2	29 [18 – 40]
Community hospital	2	75 [70 – 80]	1	14 [14 – 14]
Temporary residence	6	75 [59 – 91]	4	14 [12 – 20]
Home support				
Lives alone	188	70 [50 – 85]	173	19 [15 – 24]
Lives with someone	398	70 [50 – 80]	375	17 [14 – 22]
Lives with carer	1	35 [35 – 35]	1	34 [34 – 34]
Home care package	5	40 [25 – 55]	1	15 [15 – 15]
Walking aid pre-injury				
None	512	70 [55 – 85]	491	17 [14 – 21]
One stick	58	45 [35 – 61]	50	26 [21 – 32]
Two sticks	6	48 [30 – 65]	4	37 [18 – 59]
Frame/Rollator	13	40 [25 – 70]	4	88 [40 – 192]
Wheelchair	3	70 [45 – 95]	1	46 [46 – 46]
Walking distance pre-injury				
About house	17	55 [35 – 70]	6	29 [26 – 112]
Less than 100m	33	40 [28 – 58]	28	37 [27 – 47]
Less than 0.5 mile	58	55 [40 – 75]	52	22 [17 – 28]
More than 0.5 mile	484	70 [55 – 85]	464	17 [14 – 21]
Fracture pattern				
Weber A and B	516	70 [50 – 80]	480	18 [14 – 23]
Weber C	76	65 [45 – 79]	70	18 [15 – 23]
Treatment received				
Close contact casting	271	70 [50 – 80]	246	18 [15 – 23]
Internal fixation surgery	308	70 [55 – 80]	292	17 [14 – 22]
Other	13	55 [38 – 85]	12	20 [17 – 29]
Readmission				
No	542	70 [50 – 80]	506	18 [14 – 23]
Yes	50	63 [50 – 75]	44	18 [14 – 21]
Partial weight date present				
No	224	70 [50 – 85]	213	17 [14 – 22]
Yes	368	70 [50 – 80]	337	18 [14 – 23]
Radiological malalignment				

For peer review only

No	365	70 [50 – 80]	343	18 [14 – 23]
Yes	227	65 [45 – 80]	207	18 [15 – 23]

Missing data

Two participants had >90% missing data and were omitted from the analysis. Most predictors did not exceed 16% missing data. Most missing data occurred in the baseline EQ-5D-3L post-injury score. There were complete baseline, 6-week, and outcome (OMAS and TUG) data for 434/620 (70%) participants.

Predicting patient-reported functional outcome 6 months after ankle fracture

OMAS baseline model

TABLE 4 shows the eight baseline variables associated with 6-month OMAS and selected for the OMAS baseline model: pre-injury OMAS, alcohol consumed per week (units), post-injury EQ-5D-3L VAS, sex, walking distance, walking aid, smoking status, and health status. For instance, if all variables were kept constant, the 6-month OMAS for women was on average approximately 7 points lower than for equivalent men (p<0.001, 6-month OMAS median [IQR], 70 [50 - 80]). A 10-point difference in the pre-injury OMAS for two identical individuals led to an average difference of approximately 2 points in the 6-month OMAS. The units of alcohol consumed in a week and the post-injury EQ-5D-3L VAS had marginal (p=0.043) and non-influential (p=0.102) effects on the 6-month OMAS, respectively. People who could walk further than 0.5 miles before injury had an approximately 8-point higher OMAS than those who could not, and people who required a walking aid before injury were more likely to have an approximately 8-point lower OMAS.

For peer review only

The initial model containing all candidate predictors had an R^2 value of 0.25. The final model had an R^2 value of 0.238 and an adjusted R^2 of 0.223: the model's predictors explained approximately 22% of the outcome variation.

For peer review only

TABLE 4: Univariable analysis and the final baseline and baseline plus 6-week follow-up linear multivariable models for the 6-month OMAS score

Variable	Univariable analysis		Final multivariable baseline model		Final multivariable baseline plus 6 week follow-up model	
	β coefficient [95% CI]	p-value	β coefficient [95% CI]	p-value	β coefficient [95% CI]	p-value
Baseline						
Constant			44.34 [30.04, 58.64]	<0.001	34.62 [20.00, 49.23]	<0.001
Pre-injury OMAS score	0.48 [0.39, 0.57]	<0.001	0.22 [0.09, 0.34]	0.001	0.19 [0.07, 0.31]	0.002
Alcohol consumed per week (units)	0.34 [0.16, 0.52]	<0.001	0.14 [-0.03, 0.32]	0.102	0.23 [0.07, 0.40]	0.007
EQ-5D-3L VAS Score (post-injury)	0.18 [0.10, 0.25]	<0.001	0.08 [0.003, 0.15]	0.043		
Sex		<0.001		<0.001		0.010
Female	-8.94 [-12.82, -5.05]		-6.90 [-10.75, -3.06]		-4.99 [-8.79, -1.19]	
Walking distance*		<0.001		0.002		0.015
>0.5mile	19.10 [15.01, 23.19]		7.79 [2.97, 12.61]		5.90 [1.16, 10.64]	
Walking aid		<0.001		0.003		0.006
Yes	-20.31 [-24.95, -15.66]		-8.35 [-13.89, -2.82]		-7.60 [13.01, -2.20]	
Smoking status†		0.185		0.049		0.061
Ex-smoker	-1.57 [-7.87, 4.72]		-3.31 [-8.93, 2.30]		-3.26 [-8.73, 2.20]	
Never	1.83 [-4.33, 8.00]		0.85 [-4.69, 6.38]		0.62 [-4.76, 6.00]	
Health status‡		<0.001		0.023		0.050
Very good	-4.19 [-8.50, 0.13]		-1.79 [-5.82, 2.24]		-2.25 [-6.17, 1.66]	
Good	-13.42 [-18.13, -8.70]		-6.90, [-11.48, -2.32]		-6.50 [-10.93, -2.07]	
Fair	-20.69 [-27.41, -13.97]		-2.97 [-10.32, 4.38]		-4.48 [-11.51, 2.56]	
Poor	-23.48 [-44.30, -2.66]		5.94 [-14.25, 26.14]		2.95 [-16.61, 22.51]	
Patient age	-0.54 [-0.77, -0.31]	<0.001				
MMSE	1.21 [0.43, 2.00]	0.003				
EQ-5D-3L VAS Score (day before injury)	0.35 [0.25, 0.46]	<0.001				
EQ-5D-3L score (day before injury)	32.3 [23.07, 41.53]	<0.001				
EQ-5D-3L score (post-injury)	11.24 [4.65, 17.83]	<0.001				
Number of comorbidities	-3.58 [-4.89, -2.28]	<0.001				
Fracture pattern		0.175				
Weber C	-3.59 [-8.78, 1.60]					

For peer review only

Admitted from§		0.441				
Warden accommodation	-8.31 [-23.57, 6.96]					
Acute hospital	-10.01 [-40.38, 20.37]					
Community hospital	9.99 [-20.38, 40.37]					
Temporary residence	10.96 [-5.35, 27.26]					
Treatment received||		0.249				
ORIF	1.67 [-1.83, 5.18]					
Other	-6.33 [-16.75, 4.09]					
Home support ¶		0.003				
Live alone or with someone	26.11 [8.63, 43.59]					
Additional 6 week variables						
6-week EQ-5D-3L VAS	0.32 [0.21, 0.42]	<0.001				
Readmission to hospital						
Yes	-3.30 [-9.49, 2.89]	0.295				
Started partial weight bearing		0.575				
Yes	-1.01 [-4.54, 2.52]					
Radiological malalignment present		0.124				0.025
Yes	-2.78 [-6.33, 0.77]				-3.54 [-6.62, -0.45]	
Range of injured ankle motion Dorsiflexion	0.15 [-0.04, 0.34]	0.131			0.18 [0.007, 0.35]	0.041
Range of injured ankle motion Plantar flexion	0.14 [0.01, 0.28]	0.032			0.17 [0.05, 0.28]	0.006
6-week OMAS	0.33 [0.22, 0.44]	<0.001			0.19 [0.09, 0.30]	<0.001
6-week EQ-5D-3L score	25.69 [18.66, 32.72]	<0.001			10.21 [3.64, 16.78]	0.002

*Reference category for walking distance is "<0.5 mile"
†Reference category for smoking status is "Current smoker"
‡Reference category for health status is "Excellent"
§Reference category for admitted from is "Own home"
||Reference category for treatment received from is "CCC"
¶Reference category for home support is "live with care or has external care support"
NB: EQ-5D-3L VAS Score (post-injury) omitted from final model by backward selection and correlation with 6-week score.
OMAS: Olerud and Molander Ankle Scale; MMSE: Mini-Mental State Examination; ORIF: Open Reduction and Internal Fixation; VAS: Visual Analogue Scale

For peer review only

OMAS baseline model internal validation

After bootstrapping, the optimism-corrected R^2 performance estimate was 0.228. The model's performance on the internal validation was similar to that achieved on the original dataset, indicating no evidence for any overfitting.

OMAS baseline/6-week model

Adding the 6-week follow-up variables produced a model similar to the baseline model, minus post-injury EQ-5D-3L VAS, plus five 6-week follow-up variables: presence of radiological malalignment, range of motion in the injured ankle for dorsiflexion and plantar flexion, 6-week OMAS, and 6-week EQ-5D-3L (**TABLE 4**). A patient with radiological malalignment at 6 weeks would, on average, have a 6-month OMAS approximately 4 points lower than a patient without malalignment. Keeping all other variables constant, 20 degrees more ankle motion in both dorsiflexion and plantar flexion at 6 weeks would result in an approximately 4-point higher 6-month OMAS. The higher the 6-week OMAS and EQ-5D-3L, the greater the 6-month OMAS ($p<0.001$ and $p=0.002$, respectively).

The baseline/6-week model had an adjusted R^2 value of 0.261 which was higher than the baseline model's adjusted R^2. Including the 6-week follow-up variables explained around 4% more variation than the baseline model.

OMAS baseline/6 week model internal validation

After bootstrapping, the optimism-corrected R^2 performance estimate was 0.264, similar to that on the original dataset. Approximately 26% of the variation in the 6-

For peer review only

month OMAS was explained by the model, an increase of 3% from the baseline linear multivariable model.

In an exploratory analysis, a model using only the 6-week variables was developed. The adjusted R^2 value was 0.123, which was significantly lower than the model performance for the baseline model.

Predicting objectively measured functional outcome 6 months after ankle fracture

TUG baseline model

TABLE 5 shows the baseline variables associated with the TUG time at 6 months and included in the baseline TUG model. On average women had around 6 seconds (p<0.001) longer TUG times than men (6-month TUG median [IQR], 18 seconds [14 – 23]). Holding all other variables constant, a 10-point increase in the mini-mental state examination score led to a 2 second decrease in the predicted TUG time (p<0.001). A 10-year age difference between two otherwise identical individuals resulted in an 8-s longer predicted TUG time for the older individual (p<0.001). Those with higher post-injury EQ-5D-3L VAS were more likely to have a quicker TUG time at 6 months (p<0.001). People able to walk further than 0.5 mile before injury had a TUG time approximately 20 seconds quicker than an equivalent person who could only walk less than 0.5 mile (p<0.001). People who required a walking aid before injury were 19 s slower, on average, than those who did not. Fracture pattern and pre-injury OMAS predicted the TUG score well: a Weber C fracture pattern and low pre-injury OMAS predicted a longer TUG time.

For peer review only

The initial model containing all possible variables had an R^2 value of 0.321, indicating that all of the variables chosen for consideration in model building together explained 32% of the variation in the TUG outcome. The final baseline model had an R^2 value of 0.308 and an adjusted R^2 of 0.298, indicating that the model's predictors explained approximately 30% of the outcome variation.

For peer review only

TABLE 5: Univariable analysis and the final baseline and baseline plus 6-week follow-up linear multivariable models for the 6-month TUG score

Variable	Univariable analysis		Final multivariable baseline model		Final multivariable baseline plus 6 week follow-up model	
	β coefficient [95% CI]	p-value	β coefficient [95% CI]	p-value	β coefficient [95% CI]	p-value
Baseline						
Constant			24.86 [-0.31, 50.03]	0.05	51.72 [29.34, 74.11]	<0.001
Pre-injury OMAS score	-0.38 [-0.45, -0.30]	<0.001	-0.09 [-0.19, 0.0007]	0.05		
Alcohol consumed per week (units)	-0.24 [-0.38, -0.10]	0.001				
EQ-5D-3L VAS Score (post-injury)	-0.12 [-0.18, -0.06]	<0.001	-0.05 [-0.10, 0.006]	0.08		
Sex		<0.001		0.008		0.02
Female	5.64 [2.51, 8.78]		3.61 [0.93, 6.29]		3.30 [0.57, 6.03]	
Walking distance*		<0.001		<0.001		<0.001
>0.5mile	-19.67 [-22.80, -16.53]		-12.26 [-15.87, -8.65]		-9.90 [-13.30, -6.50]	
Walking aid		<0.001		0.001		<0.001
Yes	19.00 [15.35, 22.64]		7.67 [3.34, 11.99]		6.10 [2.35, 9.85]	
Smoking status†		0.33				0.04
Ex-smoker	-0.06 [-5.11, 4.99]				-1.26 [-5.48, 2.96]	
Never	-2.15 [-7.10, 2.81]				-3.93 [-8.07, 0.21]	
Health status‡		<0.001				
Very good	1.62 [-1.84, 5.07]					
Good	7.68 [3.90, 11.46]					
Fair	18.08 [12.70, 23.47]					
Poor	23.27 [6.60, 39.94]					
Patient age	0.79 [0.61, 0.97]	<0.001	0.36 [0.18, 0.54]	<0.001	0.37 [0.20, 0.54]	<0.001
MMSE	-2.15 [-2.75, -1.54]	<0.001	-0.89 [-1.46, -0.32]	0.002	-1.35 [-1.91, -0.79]	<0.001
EQ-5D-3L VAS Score (day before injury)	-0.35 [-0.43, -0.26]	<0.001				
EQ-5D-3L score (day before injury)	-21.22 [-28.46, -13.97]	<0.001	6.73 [-0.99, 14.44]	0.09		
EQ-5D-3L score (post-injury)	-8.48 [-13.92, -3.03]	0.002				
Number of comorbidities	3.43 [2.34, 4.46]	<0.001				
Fracture pattern		0.03		0.03		
Weber C	4.61 [0.46, 8.76]		3.97 [0.44, 7.50]			
Admitted from§		0.67				
Warden accommodation	0.82 [-11.44, 13.07]					

For peer review only

Acute hospital	6.72 [-17.66, 31.11]					
Community hospital	-0.50 [-24.89, 23.89]					
Temporary residence	-9.53 [-22.62, 3.56]					
Treatment received||		0.35				
ORIF	-0.94 [-3.75, 1.87]					
Other	4.95 [-3.41, 13.31]					
Home support¶		0.03				
Live alone or with someone	-15.40 [-29.47, -1.34]					
Additional 6 week variables						
6-week EQ-5D-3L VAS	-0.24 [-0.32, -0.16]	<0.001			-0.07 [-0.14, 0.00]	0.05
Readmission to hospital		0.06				0.03
Yes	-4.83 [-9.81, 0.15]				-4.49 [-8.60, -0.38]	
Started partial weight bearing		0.96				
Yes	0.08 [-2.77, 2.92]					
Radiological malalignment present		0.24				
Yes	-1.73 [-4.59, 1.13]					
Range of injured ankle motion Dorsiflexion	-0.005 [-0.16, 0.15]	0.95				
Range of injured ankle motion Plantar flexion	0.01 [-0.09, 0.12]	0.83				
6-week OMAS	-0.13 [-0.22, -0.04]	0.005				
6-week EQ-5D-3L score	-23.76 [-29.46, -18.06]	<0.001			-11.11 [-16.13, -6.09]	<0.001

*Reference category for walking distance is "<0.5 mile"
†Reference category for smoking status is "Current smoker"
‡Reference category for health status is "Excellent"
§Reference category for admitted from is "Own home"
||Reference category for treatment received from is "CCC"
¶Reference category for home support is "live with care or has external care support"
NB: EQ-5D-3L VAS Score (post-injury) omitted from final model by backward selection and correlation with 6-week score.
OMAS: Olerud and Molander Ankle Scale; MMSE: Mini-Mental State Examination; ORIF: Open Reduction and Internal Fixation; VAS: Visual Analogue Scale

For peer review only

TUG baseline model internal validation

After bootstrapping, the optimism-corrected R^2 performance estimate was 0.293. The model's performance on the internal validation was very similar to that achieved on the original dataset, explaining approximately 30% of the variation in the 6-month TUG score.

TUG baseline/6-week model

When the 6-week variables were included in building a model to predict the 6-month TUG time, four baseline variables were removed. The model-building process selected another baseline variable, home support, for inclusion in their place. Its inclusion led to spurious results as there was little data in one of the subcategories (lives with carer/has external care support). The variable was therefore removed from the final model. The final model also included three 6-week variables: readmission, 6-week EQ-5D-3L, and 6-week EQ-5D-3L VAS (**TABLE 5**).

If a person was readmitted in the first 6 weeks after injury, they were predicted to have a 4 seconds faster TUG time at 6 months ($p=0.04$), if all other variables were held constant. Those with a better EQ-5D-3L score at 6 weeks were more likely to have a quicker TUG ($p<0.001$). If all other variables were kept constant, an increase of 20 points in the 6-week VAS resulted in a 1.5 seconds quicker time ($p=0.04$).

The baseline/6-week data model had a greater adjusted R^2 (0.321) than the baseline data model (0.308). The model now explained 32% of the variability in TUG at 6 months, 2% more than that explained by the baseline data model.

For peer review only

TUG baseline/6-week model internal validation

After bootstrapping, optimism-corrected R^2 performance estimate was 0.314. The model's performance on the internal validation dataset was very similar to that achieved on the original dataset, explaining approximately 31% of the variation in the 6-month TUG time.

In an exploratory analysis, a model using only the 6-week variables was developed. The adjusted R^2 value was 0.109, which was lower than the model performance for the baseline model.

Sensitivity analysis

By using single imputation for the missing values in the 6-month OMAS and TUG outcomes, we assumed that the missing values occurred at random, so that variables in the dataset could predict the occurrence of missing values. To check how sensitive the four models were to this assumption, we conducted a complete-case analysis, which assumes that missing values occur completely at random.

Twenty-six participants were omitted from the OMAS models, leaving 592 participants. The same variables were selected for the baseline data model. The same variables were selected for the baseline/6-week data model, plus post-injury EQ5D-3L VAS and 'started partial weight bearing'. Both models had marginally lower adjusted R^2 values: 0.205 for the baseline data model, 2% lower than the original model, and 0.256 for the baseline/6-week data model, 0.5% lower than the original

For peer review only

model. After bootstrapping, the optimism-corrected R^2 performance estimates of 0.215 for the baseline data model and 0.268 for the baseline/6-week data model.

Sixty-eight participants were omitted from the TUG models, leaving 550 participants. The baseline data model included the same variables as before, but replaced baseline post-injury EQ-5D-3L VAS with baseline recall pre-injury EQ-5D-3L VAS and omitted pre-injury OMAS. The baseline/6-week data model included the same variables as before, plus fracture pattern and walking aid. Both models had lower adjusted R^2 values: 0.222 for the baseline data model, 8% lower than the original model, and 0.264 for the baseline/6-week data model, 6% lower than the original model. After bootstrapping, the optimism-corrected R^2 performance estimates were 0.215 for the baseline data model and 0.253 for the baseline/6-week data model.

For peer review only

DISCUSSION

A prognostic model has the potential to inform anticipated recovery, and identify people who may benefit from additional monitoring or rehabilitation. We developed and internally validated four prognostic models for 6-month outcomes after ankle fracture using recommended methods[13] and a wide range of regularly collected data on plausible prognostic factors from hospital admission and at 6-week follow-up clinics. The OMAS baseline data model included: alcohol per week (units), post-injury EQ-5D-3L visual analogue scale (VAS), sex, pre-injury walking distance and walking aid use, smoking status, and perceived health status. The baseline/6-week data model included the same baseline variables, minus EQ-5D-3L VAS, plus five 6-week predictors: radiological malalignment, injured ankle dorsiflexion and plantarflexion range of motion, and 6-week OMAS and EQ-5D-3L. The models explained approximately 23% and 26% of the outcome variation, respectively. Similar baseline and baseline/6-week data models to predict TUG explained around 30% and 32% of the outcome variation, respectively. Adding 6-week follow-up variables to baseline variables provided only a modest benefit. This benefit arguably does not outweigh the logistical issues of obtaining patient information at 6 weeks to improve prediction accuracy. The models performed similarly on the development dataset and bootstrapped internal validation datasets.

Predictive accuracy of the four models using commonly recorded clinical data to predict self-reported or objectively measured ankle function 6 months after unstable ankle fracture in adults aged over 60 years old was relatively low. As there are limitations in predictive performance, we do not recommend using these prognostic models in clinical practice as decision-making tools in isolation.

For peer review only

The variables of most predictive value across the prognostic models were sex, with females having worse outcomes than males. A cohort of 584 severe ankle sprain participants also found that females had worse outcomes.[15] Self-reported pre-injury walking distance and pre-injury walking aid use was also predictive of outcome, with those able to walk less than 0.5 miles doing and using a walking aid faring worse than those able to walk further and not needing aids. These factors are features of declining locomotor function and are likely to be related to a greater levels of frailty pre-injury.[16]

The inferences presented here cannot be extended to propose causal relationships between the predictors and outcomes, as we did not consider issues such as confounding. As the aim and design of this study prioritised optimising prognostic accuracy, predictors were considered without considering whether they could form a causal pathway or not.[17]

Comparisons with other studies that have developed prognostic models for people after ankle fracture are challenging as, to the best of our knowledge, this is the first study to focus on older adults, use a larger cohort, and internally validate the developed models to adjust for optimism. A two-centre observational study of 60 adults (mean age 49) reported that ankle dorsiflexion and fracture classification (number of malleoli injured) predicted OMAS 6 months after cast removal ($R^2 = 0.4$).[3] In a larger cohort of 150 adults (mean age 46), Lin and colleagues[7] developed a model to predict patient-reported lower limb function (Lower Extremity Functional Scale)[18] at 4 and 12 weeks after cast removal. The models were not internally

validated, but were externally validated in a separate cohort of 94 participants. Eight predictors were examined. Pain and dorsiflexion after cast removal were included in the final model, which explained about 15% of the outcome variability in the development stage and even less in the external validation.

The lack of predictive power of both our models and those in the literature indicate that functional outcomes after ankle fracture may be difficult to predict, or other prognostic factors may need to be considered to improve predictions of functional outcome. Our models may be missing informative predictors that were not captured in the AIM trial dataset. More detailed injury characteristics, such as whether fractures were uni-, bi- or tri-malleolar, were not explored. The extent of articular damage assessed by specialist imaging could provide useful prognostic information. However, any techniques using specialist equipment or personnel would hamper clinical utility for routine use and would be difficult to implement if there were resource implications. Further research is recommended to investigate whether a wider range of psychosocial and environmental factors[19] can enhance predictive accuracy. There is preliminary evidence that catastrophising and fear-avoidance are potential psychological prognostic factors that warrant further investigation in people after ankle fracture.[20]

Similar results were found for the main and sensitivity analyses of both the OMAS and TUG models. The assumption that data were missing at random was therefore plausible and conducting single imputation before model building was probably appropriate.

For peer review only

This study was limited by the use of an existing clinical trial dataset for developing the prognostic model, as the choice of potential prognostic factors were restricted to those collected in the trial. Clinical trial cohorts are usually more selective than the wider clinical population. However, the AIM trial was a pragmatic study to enhance external validity. The use of a clinical trial cohort to develop prognostic models is also not uncommon, and has resulted in robust prognostic models for other ankle injury populations that have been successfully externally validated.[21] Strengths of using this clinical trial dataset were that it had very low levels of missing data and that it reflected data that is or could be routinely collected during acute hospital admission and clinic follow-up.

CONCLUSION

We developed and internally validated prognostic models to predict functional outcomes 6 months after unstable ankle fracture in older adults using commonly recorded clinical data. These prognostic models had relatively limited accuracy in predicting self-reported or objectively measured ankle function. Other potential predictors (e.g., psychological factors such as catastrophising and fear avoidance) should be investigated.

For peer review only

Author contributions

DJK devised the study, obtained and interpreted the data and led writing the manuscript. KV analysed and interpreted the data. KW obtained the data and contributed to the study design and interpretation. DM contributed to data management and analysis. MC contributed to the study design and data interpretation. GSC oversaw the analysis plan development and the conduct of the analysis. SEL obtained the data and contributed to the study design and interpretation. All authors critically reviewed the manuscript.

Acknowledgements

We thank the AIM trial participants and collaborators. We acknowledge Jennifer A. de Beyer of the Centre for Statistics in Medicine, University of Oxford, for English language editing and William Soanes for contributing to the statistical analysis plan.

Funding

This report is independent research supported by the National Institute for Health Research (NIHR Post Doctoral Fellowship, Dr David Keene, PDF-2016-09-056). The report was supported by the NIHR Biomedical Research Centre, Oxford. Professor Lamb receives funding from the NIHR Collaboration for Leadership in Applied Health Research and Care Oxford at Oxford Health NHS Foundation Trust.

Disclaimer

The views expressed in this publication are those of the author(s) and not necessarily those of the NHS, the National Institute for Health Research or the Department of Health.

For peer review only

Ethics approval

The National Research Ethics Service Oxfordshire Committee approved use of data. All participants gave written informed consent for data to be used.

Data sharing

All data requests should be submitted to the corresponding author for consideration. Access to anonymised data may be granted following review. Exclusive use will be retained until the publication of major outputs.

Competing interests statement

There are no competing interests for any author.

For peer review only

References

1. Kannus P, Niemi S, Parkkari J, et al. Declining incidence of fall-induced ankle fractures in elderly adults: Finnish statistics between 1970 and 2014. *Arch Orthop Trauma Surg* 2016;136(9):1243-6. doi: 10.1007/s00402-016-2524-7
2. Beckenkamp PR, Lin CW, Chagpar S, et al. Prognosis of physical function following ankle fracture: a systematic review with meta-analysis. *J Orthop Sports Phys Ther* 2014;44(11):841-51, B2. doi: 10.2519/jospt.2014.5199
3. Hancock MJ, Herbert RD, Stewart M. Prediction of outcome after ankle fracture. *J Orthop Sports Phys Ther* 2005;35(12):786-92.
4. Keene D, James G, Lamb SE, et al. Factors associated with mobility outcomes in older people post-ankle fracture: An observational cohort study focussing on peripheral vessel function. *Injury* 2013;44(7):987-93. doi: http://dx.doi.org/10.1016/j.injury.2012.08.042
5. Lin CW, Moseley AM, Herbert RD, et al. Pain and dorsiflexion range of motion predict short- and medium-term activity limitation in people receiving physiotherapy intervention after ankle fracture: an observational study. *Aust J Physiother* 2009(1):31-7.
6. Keene DJ, Mistry D, Nam J, et al. The Ankle Injury Management (AIM) trial: a pragmatic, multicentre, equivalence randomised controlled trial and economic evaluation comparing close contact casting with open surgical reduction and internal fixation in the treatment of unstable ankle fractures in patients aged over 60 years. *Health Technol Assess* 2016;20(75):1-158. doi: 10.3310/hta20750

For peer review only

7. Willett K, Keene DJ, Mistry D, et al. Close Contact Casting vs Surgery for Initial Treatment of Unstable Ankle Fractures in Older Adults: A Randomized Clinical Trial. *Jama* 2016;316(14):1455-63. doi: 10.1001/jama.2016.14719

8. Olerud C, Molander H. A scoring scale for symptom evaluation after ankle fracture. *Arch Orthop Trauma Surg* 1984;103(3):190-4.

9. Mathias S, Nayak US, Isaacs B. Balance in elderly patients: the "get-up and go" test. *Arch Phys Med Rehabil* 1986;67(6):387-9.

10. Shumway-Cook A, Brauer S, Woollacott M. Predicting the probability for falls in community-dwelling older adults using the Timed Up & Go Test. *Phys Ther* 2000;80(9):896-903.

11. Makizako H, Shimada H, Doi T, et al. Predictive Cutoff Values of the Five-Times Sit-to-Stand Test and the Timed "Up & Go" Test for Disability Incidence in Older People Dwelling in the Community. *Phys Ther* 2017;97(4):417-24. doi: 10.2522/ptj.20150665

12. Lin MR, Hwang HF, Hu MH, et al. Psychometric comparisons of the timed up and go, one-leg stand, functional reach, and Tinetti balance measures in community-dwelling older people. *J Am Geriatr Soc* 2004;52(8):1343-8. doi: 10.1111/j.1532-5415.2004.52366.x

13. Steyerberg EW. Clinical prediction models : a practical approach to development, validation, and updating. New York: Springer 2009.

14. Collins GS, Reitsma JB, Altman DG, et al. Transparent Reporting of a multivariable prediction model for Individual Prognosis Or Diagnosis (TRIPOD). *Ann Intern Med* 2015;162(10):735-6. doi: 10.7326/L15-5093-2

For peer review only

15. Akacha KH, J.L. Lamb, S.E. Modelling treatment, age- and gender-specific recovery in acute injury studies. The University of Warwick Centre for Research in Statistical Methodology, 2010:1-12.

16. Middleton A, Fritz SL, Lusardi M. Walking speed: the functional vital sign. *J Aging Phys Act* 2015;23(2):314-22. doi: 10.1123/japa.2013-0236

17. Tripepi G, Jager KJ, Dekker FW, et al. Testing for causality and prognosis: etiological and prognostic models. *Kidney Int* 2008;74(12):1512-15. doi: https://doi.org/10.1038/ki.2008.416

18. Binkley JM, Stratford PW, Lott SA, et al. The Lower Extremity Functional Scale (LEFS): scale development, measurement properties, and clinical application. North American Orthopaedic Rehabilitation Research Network. *Phys Ther* 1999;79(4):371-83.

19. Kendrick D, Kelllezi B, Coupland C, et al. Psychological morbidity and health-related quality of life after injury: multicentre cohort study. *Qual Life Res* 2017;26(5):1233-50. doi: 10.1007/s11136-016-1439-7

20. Steven JL, Buer N, Samuelsson L, et al. Pain-related fear, catastrophizing and pain in the recovery from a fracture. *Scand J Pain* 2010;1(1):38-42. doi: 10.1016/j.sjpain.2009.09.004

21. Keene DJ, Schlussel MM, Thompson J, et al. Prognostic models for identifying risk of poor outcome in people with acute ankle sprains: the SPRAINED development and external validation study. *Health Technol Assess* 2018;22(64):1-112. doi: 10.3310/hta22640

For peer review only

TRIPOD Checklist: Prediction Model Development and Validation

Section/Topic	Item		Checklist Item	Page
**Title and abstract**				
Title	1	D;V	Identify the study as developing and/or validating a multivariable prediction model, the target population, and the outcome to be predicted.	Title page
Abstract	2	D;V	Provide a summary of objectives, study design, setting, participants, sample size, predictors, outcome, statistical analysis, results, and conclusions.	Abstract page
**Introduction**				
Background and objectives	3a	D;V	Explain the medical context (including whether diagnostic or prognostic) and rationale for developing or validating the multivariable prediction model, including references to existing models.	Intro section
	3b	D;V	Specify the objectives, including whether the study describes the development or validation of the model or both.	Intro section, Objectives subsection
**Methods**				
Source of data	4a	D;V	Describe the study design or source of data (e.g., randomized trial, cohort, or registry data), separately for the development and validation data sets, if applicable.	Study design subsection
	4b	D;V	Specify the key study dates, including start of accrual; end of accrual; and, if applicable, end of follow-up.	Study design subsection
Participants	5a	D;V	Specify key elements of the study setting (e.g., primary care, secondary care, general population) including number and location of centres.	Study design subsection
	5b	D;V	Describe eligibility criteria for participants.	Participants subsection
	5c	D;V	Give details of treatments received, if relevant.	Participants subsection
Outcome	6a	D;V	Clearly define the outcome that is predicted by the prediction model, including how and when assessed.	Outcome measures subsection
	6b	D;V	Report any actions to blind assessment of the outcome to be predicted.	Outcome measures, secondary outcomes section
Predictors	7a	D;V	Clearly define all predictors used in developing or validating the multivariable prediction model, including how and when they were measured.	Predictors subsection; Table 1
	7b	D;V	Report any actions to blind assessment of predictors for the outcome and other predictors.	Predictors subsection
Sample size	8	D;V	Explain how the study size was arrived at.	Statistical analysis subsection, first sentence
Missing data	9	D;V	Describe how missing data were handled (e.g., complete-case analysis, single imputation, multiple imputation) with details of any imputation method.	Statistical analysis subsection, end of first paragraph
Statistical analysis methods	10a	D	Describe how predictors were handled in the analyses.	Stats section, paragraph three.
	10b	D	Specify type of model, all model-building procedures (including any predictor selection), and method for internal validation.	Stats section, paragraph two.
	10c	V	For validation, describe how the predictions were calculated.	Stats section, paragraph five
	10d	D;V	Specify all measures used to assess model performance and, if relevant, to compare multiple models.	Stats section, end of paragraph four
	10e	V	Describe any model updating (e.g., recalibration) arising from the validation, if done.	Stats section, paragraph five
Risk groups	11	D;V	Provide details on how risk groups were created, if done.	N/A
Development vs. validation	12	V	For validation, identify any differences from the development data in setting, eligibility criteria, outcome, and predictors.	Internal validation
**Results**				
Participants	13a	D;V	Describe the flow of participants through the study, including the number of participants with and without the outcome and, if applicable, a summary of the follow-up time. A diagram may be helpful.	Participants subsection, Tables 2 and 3
	13b	D;V	Describe the characteristics of the participants (basic demographics, clinical features, available predictors), including the number of participants with missing data for predictors and outcome.	Tables 2 and 3
	13c	V	For validation, show a comparison with the development data of the distribution of important variables (demographics, predictors and outcome).	Internal validation
Model development	14a	D	Specify the number of participants and outcome events in each analysis.	Tables 4 and 5
	14b	D	If done, report the unadjusted association between each candidate predictor and outcome.	Tables 4 and 5

For peer review only

TRIPOD Checklist: Prediction Model Development and Validation

Model specification	15a	D	Present the full prediction model to allow predictions for individuals (i.e., all regression coefficients, and model intercept or baseline survival at a given time point).	Tables 4 and 5
	15b	D	Explain how to the use the prediction model.	Tables 4 and 5 contain data to calculate model
Model performance	16	D;V	Report performance measures (with CIs) for the prediction model.	Tables 2 and 5 and in text for each model
Model-updating	17	V	If done, report the results from any model updating (i.e., model specification, model performance).	In text for each model
**Discussion**				
Limitations	18	D;V	Discuss any limitations of the study (such as nonrepresentative sample, few events per predictor, missing data).	Discussion, paragraph eight
Interpretation	19a	V	For validation, discuss the results with reference to performance in the development data, and any other validation data.	Internal validation
	19b	D;V	Give an overall interpretation of the results, considering objectives, limitations, results from similar studies, and other relevant evidence.	Discussion, paragraphs one to seven
Implications	20	D;V	Discuss the potential clinical use of the model and implications for future research.	Discussion, paragraph two
**Other information**				
Supplementary information	21	D;V	Provide information about the availability of supplementary resources, such as study protocol, Web calculator, and data sets.	N/A
Funding	22	D;V	Give the source of funding and the role of the funders for the present study.	Funding statement section

*Items relevant only to the development of a prediction model are denoted by D, items relating solely to a validation of a prediction model are denoted by V, and items relating to both are denoted D;V. We recommend using the TRIPOD Checklist in conjunction with the TRIPOD Explanation and Elaboration document.

For peer review only
